# Identification of the Allosteric Binding Site for Thiazolopyrimidine on the C-Type Lectin Langerin

**DOI:** 10.1021/acschembio.2c00626

**Published:** 2022-09-26

**Authors:** Hengxi Zhang, Carlos Modenutti, Yelha Phani Kumar Nekkanti, Maxime Denis, Iris A. Bermejo, Jonathan Lefèbre, Kateryna Che, Dongyoon Kim, Marten Kagelmacher, Dennis Kurzbach, Marc Nazaré, Christoph Rademacher

**Affiliations:** † Biomolecular Systems, Max Planck Institute of Colloids and Interfaces, Am Mühlenberg 1 14424 Potsdam, Germany; ‡ Department of Biology, Chemistry, and Pharmacy, Freie Universität Berlin, Takustrasse 3, 14195 Berlin, Germany; § Leibniz Forschungsinstitut für Molekulare Pharmakologie (FMP), Robert-Roessle-Strasse 10, 13125 Berlin, Germany; ∥ Berlin Institute of Health (BIH), Anna-Louisa-Karsch-Strasse 2, 10178 Berlin, Germany; ⊥ Department of Pharmaceutical Sciences, University of Vienna, Josef-Holaubek-Platz 2, 1090 Vienna, Austria; # Department of Microbiology and Immunobiology, Max F. Perutz Laboratories, University of Vienna, Dr.-Bohr-Gasse 9, 1030 Vienna, Austria; ∇ Faculty of Chemistry, Institute of Biological Chemistry, University of Vienna, Währinger Straße 38, 1090 Vienna, Austria; ○ Vienna Doctoral School of Pharmaceutical, Nutritional and Sport Sciences (PhaNuSpo), University of Vienna, Universitätsring 1, 1010 Vienna, Austria; ◆ Departamento de Química Biológica, Facultad de Ciencias Exactas y Naturales, C1428EHA Buenos Aires, Argentina; ¶ Instituto de Química Biológica de la Facultad de Ciencias Exactas y Naturales (IQUIBICEN), CONICET, C1428EHA Buenos Aires, Argentina; ● Doctoral School in Chemistry (DoSChem), University of Vienna, Währingerstr. 42, 1090 Vienna, Austria

## Abstract

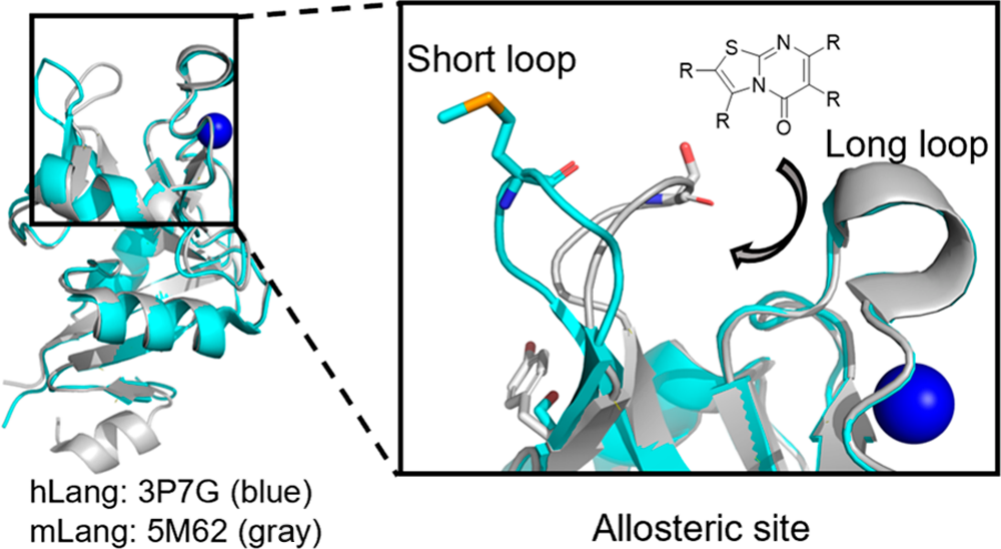

Langerin is a mammalian C-type lectin expressed on Langerhans cells in the skin. As an innate immune cell receptor, Langerin is involved in coordinating innate and adaptive immune responses against various incoming threats. We have previously reported a series of thiazolopyrimidines as murine Langerin ligands. Prompted by the observation that its human homologue exhibits different binding specificities for these small molecules, we report here our investigations to define their exact binding site. By using structural comparison and molecular dynamics simulations, we showed that the nonconserved short loops have a high degree of conformational flexibility between the human and murine homologues. Sequence analysis and mutational studies indicated that a pair of residues are essential for the recognition of the thiazolopyrimidines. Taking solvent paramagnetic relaxation enhancement NMR studies together with a series of peptides occupying the same site, we could define the cleft between the short and long loops as the allosteric binding site for these aromatic heterocycles.

Langerin is a pattern recognition receptor that is primarily expressed by Langerhans cells in the skin and mucosa.^1^ While human Langerin (hLang) has a restricted expression pattern, in mice it can be found on various dendritic cells and tissues, not limited to the skin.^2^ The two homologues have evolved to recognize different incoming threats based on the carbohydrate signatures and show remarkable differences in their specificity for bacterial polysaccharides.^3,4^ Langerin binds to pathogen-associated carbohydrates, which in turn initiates the uptake of the exogenous particle and promotes the cross-presentation of the antigen for the subsequent immune response.^5^ Accordingly, Langerin plays a key role in the transition from innate to adaptive immunity, rendering the receptor an attractive target for targeted delivery for immunotherapeutic approaches.^6,7^ Therefore, ligands with high selectivity for Langerin would be highly valuable for understanding its biology as well as for applications in targeted drug delivery.

On the one hand, we previously reported the development of a carbohydrate-based glycomimetic targeting ligand for hLang that can be applied for ex vivo delivery of liposomal encapsulated as well as directly functionalized protein cargo.^7^ On the other hand, we had developed a set of thiazolopyrimidine derivatives as selective non-carbohydrate-based ligands for murine Langerin (mLang).^8^ Thiazolopyrimidine derivatives were suggested to bind to mLang at an unidentified secondary binding sites with micro- to millimolar affinity and were proposed to block carbohydrate recognition allosterically.^8^ However, the location of the allosteric site was not identified, hindering further rational development of these and other ligands.

Some of the previously identified allosteric thiazolopyrimidine inhibitors for mLang^8^ showed highly selective binding to mLang over hLang. Comparing the binding affinities of the same compounds but different homologue targets give an estimate of whether the respective binding site is conserved. Therefore, we first performed titrations of thiazolopyrimidine-5-one derivatives with both hLang and mLang using ^1^H–^15^N heteronuclear single-quantum coherence (HSQC) NMR spectroscopy. The binding affinities of several compounds vary widely on both homologues (Table 1). For example, compounds **2**, **5**, **6**, and **7** showed no detectable binding to hLang while exhibiting micromolar affinity to mLang. However, compound **1** bound hLang with more than 10-fold higher affinity compared to mLang (*K*_D,mLang_ = 1800 ± 600 μM, *K*_D,hLang_ = 130 ± 40 μM). This behavior indicates that the ligands bind to either a nonconserved site of the protein or a completely different site on each homologue.

**Table 1:**
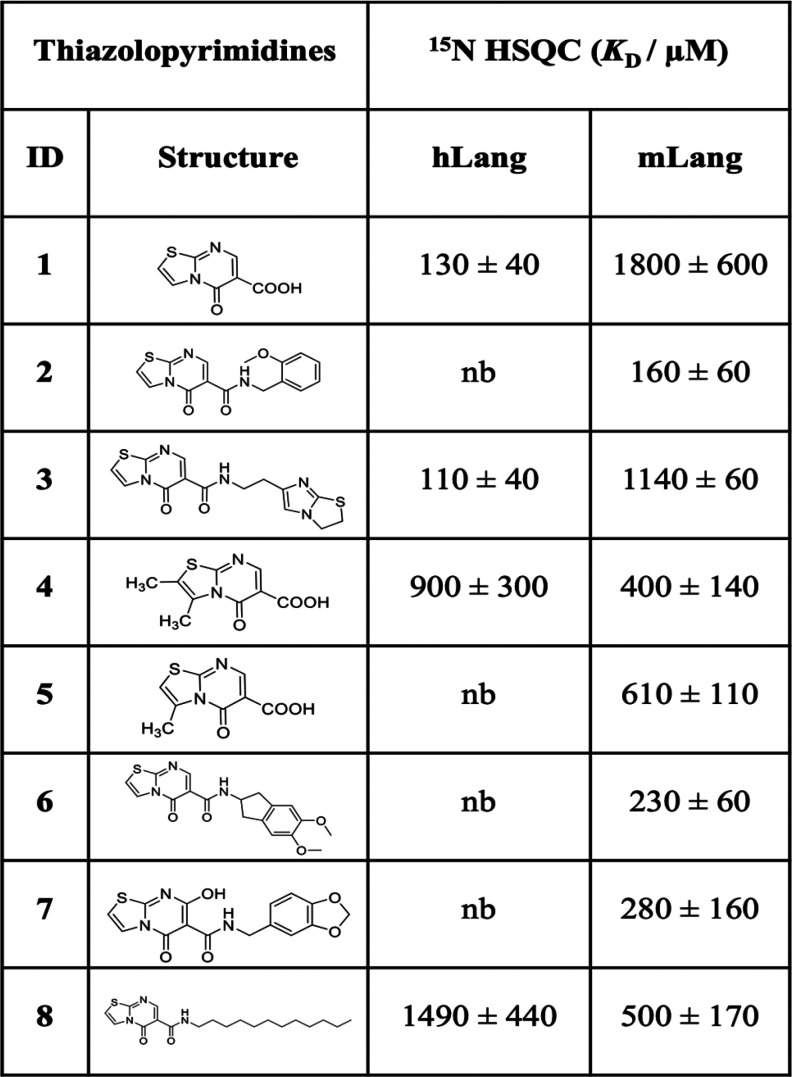
Binding Affinities of Thiazolopyrimidine Derivatives for mLang and hLang

To explore where the potential ligand binding sites are located, we used the web-server-based prediction tool PARS (Protein Allosteric and Regulatory Sites) for both hLang and mLang. PARS initially detects pockets on the input X-ray structure, followed by normal mode analysis (NMA) of the protein in the absence and the presence of a dummy ligand binding to the respective pockets.^9−13^ For both structures (hLang, PDB entry 3C22; mLang, PDB entry 5K8Y), three equivalent candidate allosteric sites were predicted (Tables S1 and S2 and Figures S1 and S2). Pocket 1 is located close to the carbohydrate binding site (CBS) and the associated Ca^2+^ binding site in the cleft between the short loop and the long loop. In contrast, pockets 2 and 3 are remote from the CBS and located in the cleft between α-helix 2 and the lower β-sheet.

To investigate the structural difference of pockets 1, 2, and 3 between the two homologues, we performed a structural alignment between hLang and mLang and found that the backbone structures are highly similar (RMSD = 0.46 Å). However, the orientation of the short loop differed significantly (RMSD = 2.19 Å) (Figure 1). While the short loop in hLang tends to point toward the outer side of the cleft between the short and long loops, forming an open state, the short loop of mLang is oriented inward toward the long loop, resembling a closed state. To deepen our understanding of this conformational difference, we conducted molecular dynamics (MD) simulations to estimate the stability of the observed conformations using the distance between the amide nitrogen of S263 (M260 in hLang) and the carbonyl oxygen of N294 (N291 in hLang) in the short loop as a measure (Figure S3). In line with the described open and closed conformations, two distinct preference states of the short loop for hLang and mLang were observed. While mLang favors the closed state in the X-ray structure, with the open state occurring with 18% occupancy in total (Figure S3), the short loop of hLang showed only 1–2% occupancy of the closed state, suggesting the open state of hLang to be the dominant conformation. Therefore, the different conformations of the short loop in the two homologues indicated that pocket 1 is different between hLang and mLang.

**Figure 1:**
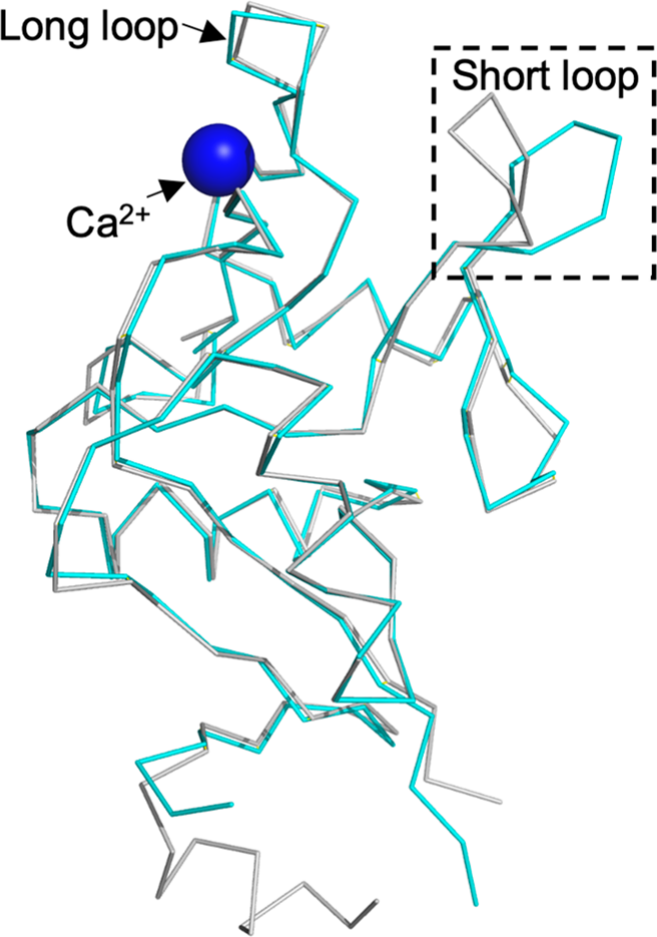
Structural difference between mLang and hLang. Ribbon representations of conformational differences in the short loop between mLang (gray) and hLang (blue) are shown. The dashed box shows the main conformational difference between the two homologues located in the short loop region. The hLang structure is from PDB entry 3P7G. The mLang structure is from PDB entry 5M62.

This analysis clearly indicates that the short loop behaves differently in hLang and mLang. Therefore, we investigated the sequence difference between the two homologues. First, we performed multiple sequence alignment (MSA) of the short loop regions of 28 Langerin homologues and calculated the positional sequence conservation. This revealed lower evolutionary conservation of two amino acids in the short loop compared to the rest of the sequences (Figures 2 and S4). A pattern arises from the comparison of central residues of the short loop and their complementary counterparts making interloop interactions. For example, the smaller sized S/T (S263 in mLang) in this position is more likely to be complemented with a large amino acid such as Y/H/F (Y268 in mLang) (Figures 2 and S4). On the other hand, the large-sized residue M (M260 in hLang) in this position is more likely to be complemented with a small amino acid S (S265 in hLang).

**Figure 2:**
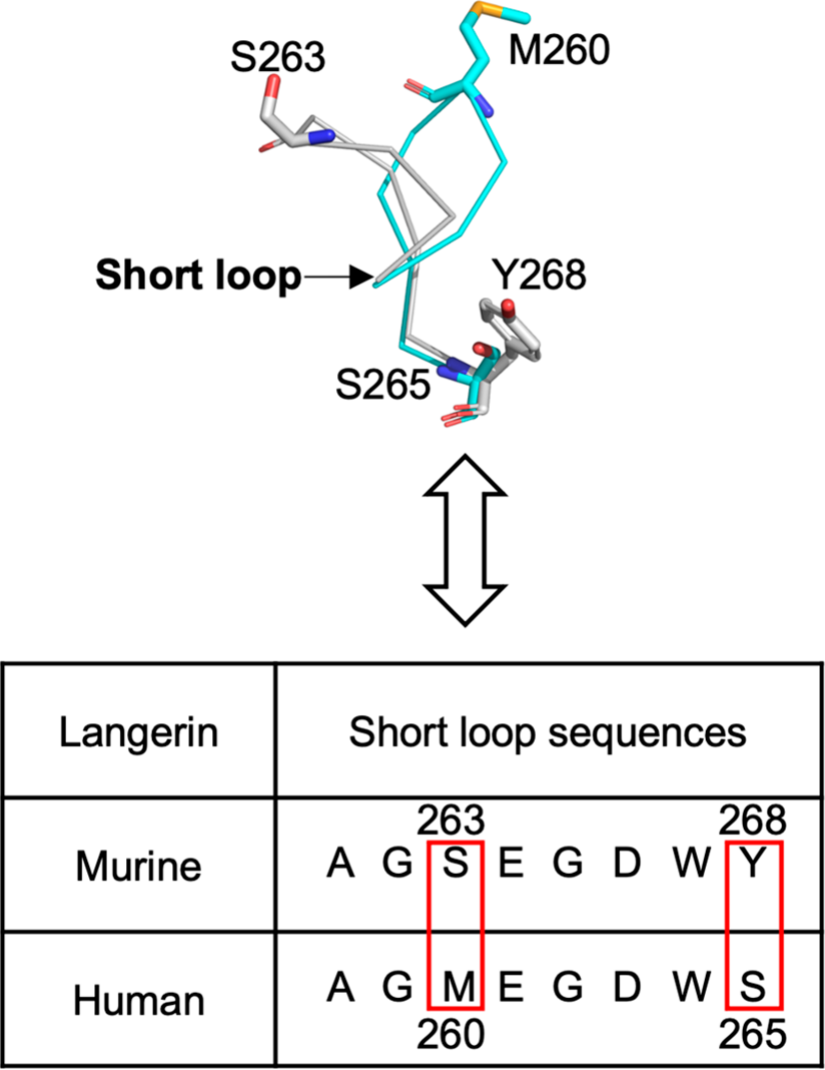
Double mutations in central short loop residues for mLang and hLang change the binding specificity of the thiazolopyrimidine derivatives. A comparison of the central short loop residues between hLang and mLang in structure and sequence is presented. The short loop shows a highly nonconserved property, and the most striking differences between the two species are S263 and Y268 in mLang versus M260 and S265 in hLang. The hLang structure is from PDB entry 3P7G (blue). The mLang structure is from PDB entry 5M62 (gray).

We hypothesized that these differences in the short loop conformation could account for the observed specificity of thiazolopyrimidine derivatives. To test this, we expressed two double mutants, exchanging the central short loop residues and their complementary amino acids, yielding hLang M260S-S265Y and mLang S263M-Y268S. We then selected two thiazolopyrimidine derivatives **1** and **2**, one with better selectivity for hLang than for mLang and vice versa, to assess whether these two double mutants could alter the compound binding specificity.

We first confirmed that compounds **1** and **2** share the same binding site in mLang (Figure S5) and then measured the binding affinity for both mutants with thiazolopyrimidine derivatives **1** and **2** by using ^1^H–^15^N HSQC NMR spectroscopy (Table 2 and Figures S8–S14). Intriguingly, comparative titrations revealed reversed specificity of the compounds for the mutants compared to the WT: compound **1** showed a 3-fold decrease in affinity to hLang M260S-S265Y (*K*_D_ = 340 ± 220 μM), while the affinity for mLang S263M-Y268S increased 3-fold (*K*_D_ = 480 ± 340 μM) (Table 2). Vice versa, the binding affinity of compound **2** to hLang M260S-S265Y increased from not detectable to the high-micromolar range (*K*_D_ = 450 ± 280 μM), whereas the binding affinity to mLang S263M-Y268S decreased by 10-fold (*K*_D_ = 1740 ± 160 μM) (Table 2). Hence, we confirmed that the nonconserved central short loop residues and their complementary counterparts can significantly change the thiazolopyrimidine binding specificity between mLang and hLang.

**Table 2:**
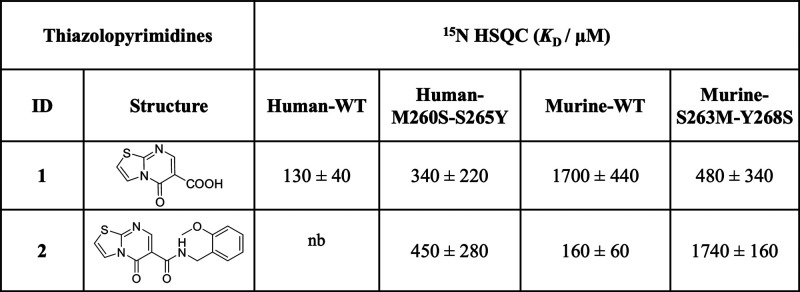
Comparison of Binding Affinities of Thiazolopyrimidine Derivatives with Wild-Type and Mutant hLang and mLang

To further support that pocket 1 is the ligand binding site, we used TEMPOL-induced solution paramagnetic relaxation enhancement (sPRE) in combination with ^15^N HSQC NMR spectroscopy. sPRE makes use of the TEMPOL-induced line broadening effect on the resonances of the protein, which is dependent on the solvent accessibility of the respective residues.^14−16^ Consequently, sPRE NMR allows the mapping of the ligand binding site by monitoring the protection of the binding site residues by a ligand (Figures 3A and S6). After having assigned the backbone ^1^H–^15^N chemical shift (Figure S15), we tested mannose as an endogenous ligand using the mLang carbohydrate recognition domain (CRD) and mapped the ΔsPRE effects onto the structure as a control. This revealed inaccessibility of residues by TEMPOL in the CBS in proximity to the Ca^2+^ sites, such as N290, N291, and A292 (Figure S7). Notably, N290 is located in the well-characterized EPN motif. Additionally, residues being highly solvent-accessible following the binding of mannose were found to be located in the short loop (E264 and G265) and long loop (G293 and N295), indicating that mannose binding could alter their accessibility. Furthermore, this suggests structural changes in both the short and long loops upon carbohydrate binding. Similar observations have been previously reported for E-Selectin, where large conformational changes in the long loop upon interaction with sialyl LewisX and a glycomimetic in the Ca^2+^ binding site were identified.^17^

**Figure 3:**
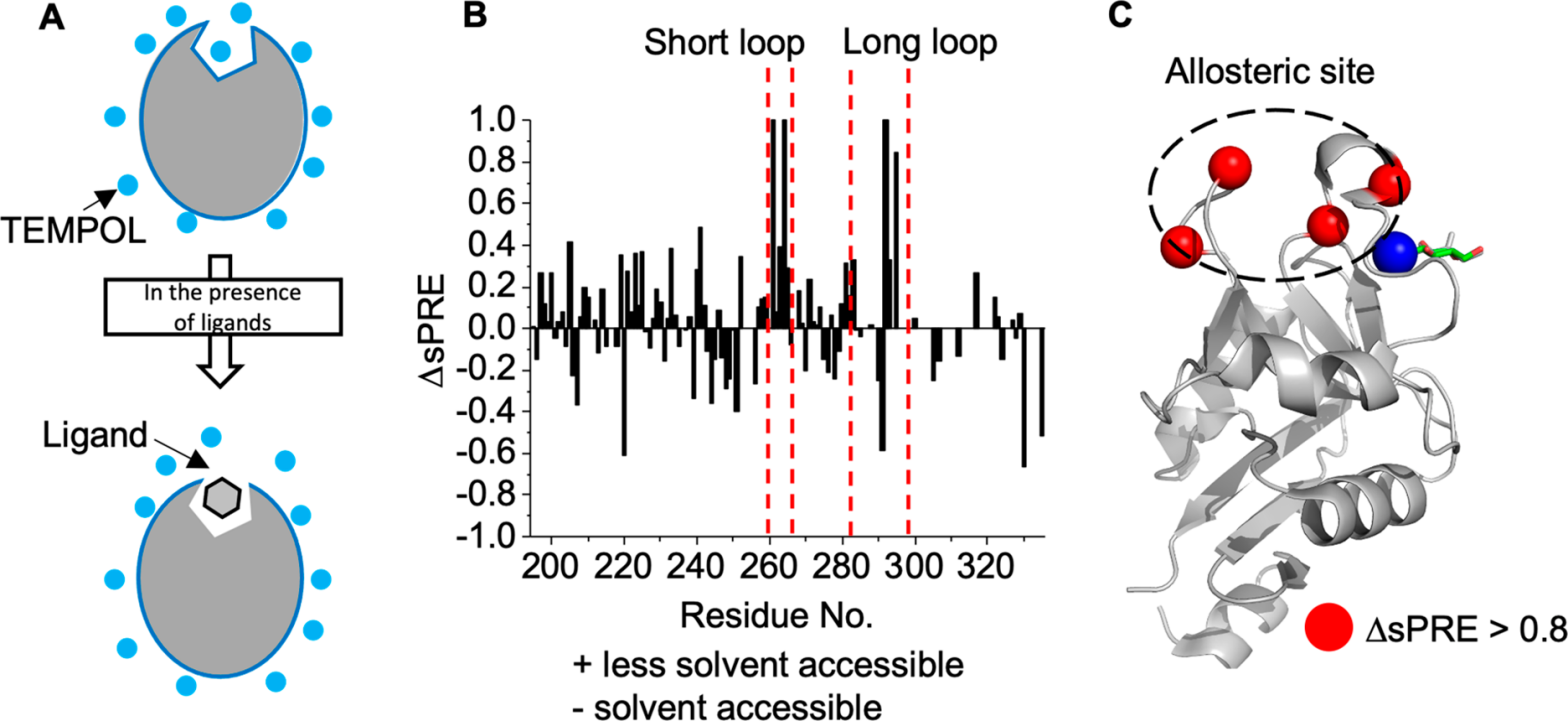
Allosteric binding site confirmation by sPRE. (A) Schematic description of the sPRE method to identify the ligand binding site. (B) sPREs were obtained from the signal amplitudes in ^1^H–^15^N HSQC NMR spectra at concentrations of 15 mM of the soluble paramagnetic agent TEMPOL. The positive value in the histogram represents the less solvent-accessible site upon ligand binding and negative values the opposite. (C) TEMPOL hot spot mapping on the mLang structure (PDB entry 5M62). The residues A261 and E264 in the short loop and A292 and E296 exhibiting ΔsPRE > 0.8 are labeled as red-colored sphere with size 0.5 on the cartoon presentation of the protein. The allosteric binding site is located in the cleft between the short and long loops, which overlapped with pocket 1 predicted by PARS, and is marked with black dashed ellipse.

Next, we mapped the binding site of compound **2** with mLang. Residues of the short loop (A261 and E264) and the long loop (A292 and E296) located in proximity to the predicted pocket 1 showed the highest ΔsPRE values, suggesting direct interaction of **2** with the pocket between the short and long loops (Figure 3B,C). On the basis of this result and the fact that compounds **1** and **2** can compete, we therefore conclude that the thiazolopyrimidine derivatives **1** and **2** bind at the same site.

This cleft has been reported earlier to harbor a peptide from a C-terminal strep tag in X-ray structures (PDB entry 3P7H) (Figure 4A).^18^ After closer inspection, we found that E261 and G259 in the short loop form hydrogen bonds with tryptophan and serine in the strep tag. Additionally, Q288 and H294 in the long loop and K256 form hydrogen bonds with alanine and tryptophan of the strep tag. Comparison of the peptide with our thiazolopyrimidine derivatives indicated similarity in their aromatic scaffolds, suggesting similar binding activity (Figure 4B).

**Figure 4:**
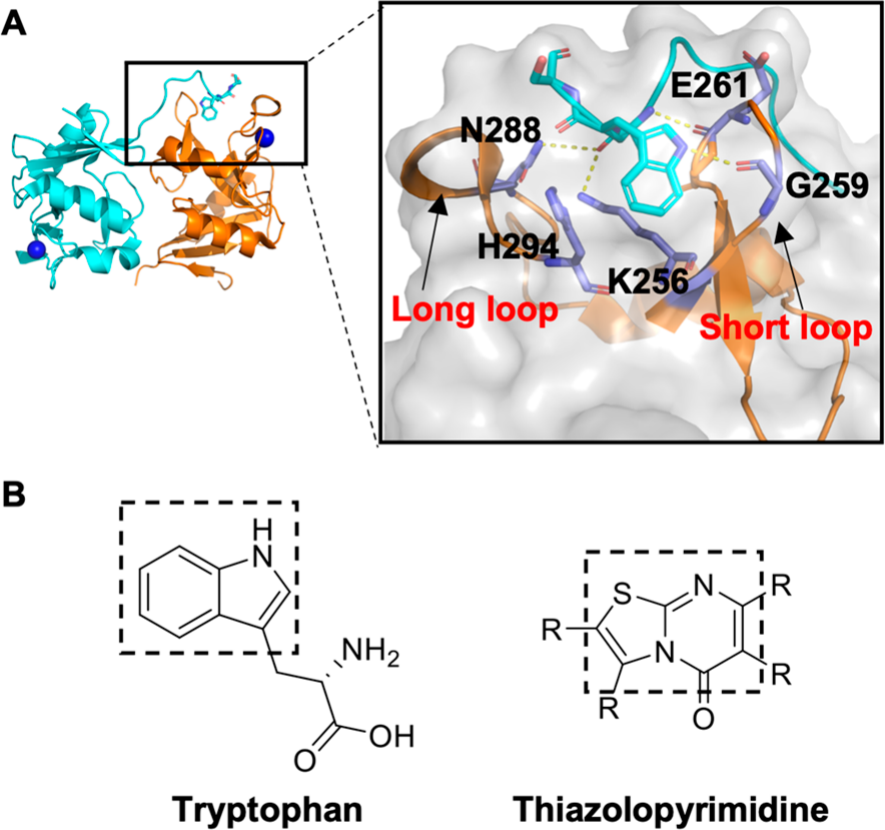
Tripeptides in strep tag bind to the cleft between the short and long loops. (A) Cartoon representation of the X-ray structure of the hLang CRD (PDB entry 3P7H).^18^ A C-terminal strep tag peptide binds to the cleft between the short and long loops (left). The interactions of the peptide with the short and long loops are magnified (right). (B) Structure presentation of tryptophan and the similar structure of the thiazolopyrimidine inhibitor.

To further verify the binding to Langerin, we designed and synthesized six tryptophan-containing peptides based on the tripeptide in the strep tag (PDB entry 3P7H) (Table S3). First, we conducted single-point titrations by adding 1 mM peptide to the protein and measured ^1^H–^15^N HSQC NMR spectra to give a qualitative ranking of the peptides. From this, we noticed that all of the peptides induced similar low chemical shift perturbations (CSPs) in the spectrum of hLang, indicating low binding affinity. Among them, the **SAWS** peptide showed the highest CSPs compared to the other peptides (Figure S16). In the case of mLang, we observed a significant change in the chemical shift of A261 for each peptide bound to mLang. Especially **SAWS**, **SSWS**, and **EPSSWS** induced strong perturbations (between 0.05 and 0.1 ppm) (Figure S17), suggesting that A261 is highly affected by peptide binding. Notably, A261 is located in the short loop region.

Next, we performed full titrations of **AWS** and **SAWS** with hLang and **AWS**, **SAWS**, **SSWS**, and **EPSSWS** with mLang. As expected, **AWS** showed similar binding affinity for both hLang and mLang (2400 ± 940 and 1600 ± 300 μM). Other than that, the larger peptides, for example **SAWS**, showed high-micromolar affinity for mLang and hLang (Table S3 and Figure S18), which is comparable to the observed binding affinity of the thiazolopyrimidine derivatives. Overall, these data, combined with the analysis of the X-ray structure, suggest that our designed peptides bind to Langerin in the millimolar to submillimolar range at the cleft between the short and long loops, which was also proposed to be the binding pocket for the aromatic heterocyclic core of the thiazolopyrimidines.

In conclusion, we previously found that Langerin is regulated by an intradomain allosteric network coupling the mobilities of the short and long loops, which in turn can modulate the Ca^2+^ affinity.^19^ In this arrangement, the cleft between the short and long loops harbors a pH sensor H294 that perturbs the Ca^2+^ affinity by affecting the hydrogen-bonding network between the loops upon protonation.^20^ In addition, we developed a series of thiazolopyrimidines as specific ligands for mLang following a fragment screening approach.^8^ We found that thiazolopyrimidines that bind to a secondary site are allosteric inhibitors of the carbohydrate site.^8^ However, we could not define the exact location of this secondary site. In this report, we have identified the cleft between the short and long loops as the secondary binding site for thiazolopyrimidines by a series of experiments using site-directed mutagenesis, MD simulations, and sPRE NMR.

Our findings further indicate the existence of two distinct classes of Langerin homologues based on distinct residue patterns in the short loop. We found that two nonconserved short loop residues form two distinct patterns across 28 mammalian species. In one pattern, a small amino acid (S/T) is complemented by a large residue (Y/H/F) located in another nonconserved site. In the other case, a large residue (M) is complemented by a small residue (S) (Figures 2 and S4). These two patterns correspond to two conformations of the short loop. Using mutagenesis, we were able to convert one pattern into the other, resulting in a switch of the ligand-binding specificity between hLang and mLang. This finding indicates that the two nonconserved short loop residues directly determine the thiazolopyrimidine binding specificity.

It is intriguing that this cleft between the two loops is also involved in peptide recognition. Besides harboring a peptide from a strep tag (PDB entry 3P7H), this cleft has also been reported to bind itself in an interdomain recognition enabling Birkbeck granule formation (PDB entry 7WZ8).^21^ Therefore, this secondary site is also involved in the biological function of Langerin. How this recognition is promoted in the absence of Ca^2+^ in the Birkbeck granules is a matter of future research. In other C-type lectins, this site harbors additional Ca^2+^ ions. For example, DC-SIGN and DC-SIGNR have two Ca^2+^ ions in this cleft, suggesting that this allosteric site may have different functions in other C-type lectins.

## Supporting Information Available

Supplementary methods (protein expression and purification, ^1^H–^15^N NMR, STD competition NMR, sPRE NMR, MD simulation, compound synthesis details), Tables S1–S3, and Figures S1–S18 (PDF)
