# Supplementary material for: Identification of the Allosteric Binding Site for Thiazolopyrimidine on the C-Type Lectin Langerin
Source: ACS Chem Biol. 2022 Sep 26;17(10):2728–33. doi: 10.1021/acschembio.2c00626 (PMC9594047; doi:10.1021/acschembio.2c00626)
Supplement: Supplementary file 1 — cb2c00626_si_001.pdf [file cb2c00626_si_001.pdf]

Supplementary information

for

"Identification of the allosteric binding site for thiazolopyrimidine on C-type lectin Langerin"

Hengxi Zhang[1,2,5,6,8], Carlos Modenutti[1,9,10], Yelha Phani Kumar Nekkanti[3,4], Maxime Denis[5,6], Iris A. Bermejo[5,6], Jonathan Lefèbre[5,6,8], Kateryna Che[7], Dongyoon Kim[1,5,6], Marten Kagelmacher[1,2], Dennis Kurzbach[7], Marc Nazaré[3,4], Christoph Rademacher[1,2,5,6]*

[1]Max Planck Institute of Colloids and Interfaces, Biomolecular Systems, Am Mühlenberg 1, 14424 Potsdam, Germany

[2]Department of Biology, Chemistry, and Pharmacy, Freie Universität Berlin, Takustrasse 3, 14195 Berlin, Germany

[3]Leibniz Forschungsinstitut für Molekulare Pharmakologie (FMP), Robert-Roessle-Strasse 10, 13125 Berlin, Germany

[4]Berlin Institute of Health (BIH), Anna-Louisa-Karsch-Strasse 2, 10178 Berlin, Germany

[5]Department of Pharmaceutical Sciences, University of Vienna, Josef-Holaubek-Platz 2, 1090 Vienna, Austria

[6]Department of Microbiology and Immunobiology, Max F. Perutz Laboratories, University of Vienna, Dr.-Bohr-Gasse 9, 1030 Vienna, Austria

[7]Faculty of Chemistry, Institute of Biological Chemistry, University of Vienna, Währinger Strasse 38, 1090 Vienna, Austria

[8]Vienna Doctoral School of Pharmaceutical, Nutritional and Sport Sciences (PhaNuSpo), University of Vienna, Universitätsring 1, 1010 Vienna, Austria

[9]Departamento de Química Biológica, Facultad de Ciencias Exactas y Naturales, C1428EHA Buenos Aires, Argentina,

[10]Instituto de Química Biológica de la Facultad de Ciencias Exactas y Naturales (IQUIBICEN), CONICET, C1428EHA Buenos Aires, Argentina

*Corresponding author: christoph.rademacher@univie.ac.at

Table of Contents

Supplementary Methods S5

Protein expression and purification S5

^{1}H-^{15}N HSQC NMR measurements and backbone resonance assignment S6

STD competition NMR measurements S7

Solution paramagnetic relaxation enhancement (sPRE) experiments S7

MD simulation S8

General experimental details for compounds in Table 1 .... S9

General experimental details for compounds in Table S3 .... S16

Supplementary Tables S35

Table S1. Potentially allosteric pockets predicted by PARS for hLang. S35

Table S2. Potentially allosteric pockets predicted by PARS for mLang. S36

Table S3. Peptides binding affinity for mLang and hLang. S37

Supplementary Figures S38

Figure S1. Potentially allosteric pockets predicted by PARS for hLang. S38

Figure S2. Potentially allosteric pockets predicted by PARS for mLang. S39

Figure S3. Measurement of distance between the short and long loop in mLang and hLang. S40

Figure S4. Multiple sequence alignment (MSA) and sequences conservation analysis in the short loop. S41

Figure S5. STD competition NMR experiments confirm that compound 1 and 2 are located in the same binding site. S42

Figure S6. The intensity ratio of each amino acid residue between diamagnetic and paramagnetic effect of mLang CRD in the presence of a different concentration of TEMOL. S43

Figure S7. Mannose-binding site confirmation of mLang by solution paramagnetic relaxation enhancement (sPRE) experiments. S44

Figure S8. ^{15}N HSQC CSP fingerprint of compound 1 binding with hLang CRD wild type. S45

Figure S9. ^{15}N HSQC CSP fingerprint of compound 1 binding with hLang CRD-M260S-S265Y mutant. S46

Figure S10. ^{15}N HSQC CSP fingerprint of compound 2 binding with hLang CRD-M260S-S265Y mutant. S47

Figure S11. ^{15}N HSQC CSP fingerprint of compound 1 binding with mLang CRD wild type. ... S48

Figure S12. ^{15}N HSQC CSP fingerprint of compound 2 binding with mLang CRD wild type. ... S49

Figure S13. ^{15}N HSQC CSP fingerprint of compound 1 binding with mLang CRD-S263M-Y268S. S50

Figure S14. ^{15}N HSQC CSP fingerprint of compound 2 binding with mLang CRD-S263M-Y268S. S51

Figure S15. ^{1}H-^{15}N HSQC NMR spectrum with assigned backbone and side-chain resonances of recombinant mLang CRD construct His tag. S52

Figure S16. CSPs of 1 mM peptides binding with hLang CRD WT. S53

Figure S17. CSPs of 1mM peptides binding with mLang CRD WT. S54

Figure S18. Binding affinity of peptides to hLang and mLang by ^{15}N HSQC NMR. S55

References S56

Supplementary Methods

Protein expression and purification

hLang and mLang CRD wild type and all the mutants, ranging from amino acids 193 - 328 and 194 - 331, were cloned from a codon-optimized Langerin gene for bacterial expression (GenScript, Piscataway, NJ, USA) into a pET-28a vector (GenScript, Piscataway, NJ, USA) carrying an N-terminal His-tag and a T7 promoter. All constructs were expressed in *E. coli* BL21 (ThermoFisher Scientific, Waltham, MA, USA) in LB medium or in M9 medium at 37 °C. Protein expression was induced by adding 1 mM IPTG (isopropyl β-D-1-thiogalactopyranoside) at an OD_{600} between 0.7 - 1 and were grown overnight at 37 °C. Bacteria were harvested by centrifugation at 4000 g for 30 mins. Cells were lysed in lysis buffer (50 mM Tris, 150 mM NaCl, 10 mM $MgCl_2$, 0.1% Tween-20, pH 8) with 1 mg/mL lysozyme (Sigma Aldrich, St. Louis, MO, USA) and 100 µg/mL DNase I (Applichem, Darmstadt, Germany) for at least 3 h at room temperature or 4 °C overnight or sonicated 1 h with amplitude 55%. Inclusion bodies were harvested by centrifugation at 15000 g for 30 mins.

Inclusion bodies were washed twice with lysis buffer and ultrapure water to remove cell debris and soluble proteins. Then inclusion bodies were denatured with 20 mL of denaturation buffer (6 M guanidinium hydrochloride in 100 mM Tris (pH 8)) with 0.01% β-mercaptoethanol for at least 1 h at 37 °C, shaking at 220 rpm. The supernatant was slowly diluted 1:10 with CTL refolding buffer (0.4 M L-arginine in 50 mM Tris, 20 mM NaCl, 0.8 mM KCl, pH 8.3) with 1 mM reduced glutathione (GSH) and 0.1 mM oxidized glutathione (GSSG) while stirring at 4 °C for at least 48 h after centrifugation (15,000 x g, 90 min, 4 °C). The refolded protein solution was dialyzed against 5 L TBS buffer (50 mM Tris, 150 mM NaCl, 5 mM $CaCl_2$) and then centrifuged to remove precipitated protein (15,000 x g, 90 min, 4 °C). The supernatant was purified by Ni-NTA agarose

affinity chromatography (Thermo Fisher) and the fractions of the elution were pooled and dialyzed against MES buffer (25 mM MES, 40 mM NaCl, 5 mM $CaCl_2$, pH 6). Precipitated protein was removed by centrifugation (15,000 x g, 90 min, 4 °C) and the supernatant was used for experiments. We then concentrated protein with 5 - 6 mg/mL as the final concentration.

^{1}H-^{15}N HSQC NMR measurements and backbone resonance assignment

NMR assignment and ^{15}N-HSQC NMR for CSP plot and K_D calculations were performed on a 700 MHz Bruker Advance III spectrometer (Bruker, Billerica, MA, USA) equipped with a PRODIGY probe. For mLang CRD backbone assignment, we performed a series of standard triple resonance experiments HNCA, HNCACB, HN(CO)CA and HN(CO)CACB as Hanske et al. described.[1] Data was analyzed by using CcpNmr 2.5.1.[2] In total, 88% of the residues were unambiguously assigned. Most of the unassigned residues are prolines or residues in the mobile loops near the CBS, as reported for similar protein for example human Langerin.[1] For the sample preparation, we used 300 µM ^{15}N and ^{13}C labeled protein, which is in 25 mM MES, 40 mM NaCl, 5 mM $CaCl_2$, pH 6 containing 100 µM DSS, 0.05% NaN_3, and 10% D_2O buffer. ^{1}H–^{15}N HSQC NMR titrations experiments were performed with 100 µM of Langerin CRD containing 50 µM 4,4-dimethyl-4-silapentane-1-sulfonic acid (DSS) as an internal reference, and 10% D_2O. 128 increments and 4 - 16 transients were recorded at 298 K, depending on the protein concentration and the machine implemented. NMR titration data were processed and analyzed, as previously done in Hanske et al. described (2016).[1] The protein sample was added stepwise for titration of the thiazolopyrimidine derivatives or peptides until the most solubility tolerance was reached as the final concentration. Step size according to expected K_D varied.

STD competition NMR measurements

STD competition NMR was performed on 500 MHz Bruker (Bruker, Billerica, MA, USA) equipped with a PRODIGY probe. STD competition NMR measurements were performed with 40 μM of mLang ECD in deuterated tris buffer (pH 7.8) containing 25 mM Tris-d_{11}, 150 mM NaCl, 5 mM $CaCl_2$ and 50 μM trimethylsilylpropanoic acid (TSP) as an internal reference. 2 mM compound **1** are used as a reporter molecule. Spectra were recorded in the absence and in the presence of 300 μM compound **2**. For each spectrum, 512 scans were recorded in 5 mm sample tubes at sample volumes of 500 μL. Saturation time t_{sat} was set to 2.0 s by implementing a train of Gauss pulses. The frequencies of irradiation on-resonance and off-resonance were set at 0.0 ppm and 80.0 ppm. The relaxation delay d_1 was set at 2.0 s, and the acquisition time t_{acq} was set to 1.7 s. STD NMR spectra were analyzed in MestReNova 14.1 (Mestrelab Research, Santiago de Compostela, Spain).

Solution paramagnetic relaxation enhancement (sPRE) experiments

Solution paramagnetic relaxation enhancement (sPRE) experiments were performed on a 700 MHz Bruker Advance III spectrometer (Bruker, Billerica, MA, USA) equipped with a PRODIGY probe. Paramagnetic NMR sample contains 200 μM final Langerin CRD concentration, 25 mM MES, 50 μM DSS, 40 mM NaCl, 5 mM $CaCl_2$, 15 mM TEMPOL (Sigma Aldrich, St. Louis, MO, USA), and pH is 6. The TEMPOL concentration in 15 mM produced significant cross-peak attenuations in 2D spectra with a favorable S/N ratio. We used double amount of ascorbate (30 mM) as a control experiment. The concentration of compounds **2** is 2 mM. The ^{1}H–^{15}N HSQC diamagnetic and paramagnetic spectra were obtained with a sequence of hsqcetfpf3gpsi2 (Bruker pulse sequence), 256 increments and 48 scans over 2048 data points. NMR data were processed and analyzed by CCPN Version 3.1.0. The following calculation of the ΔsPRE can be described as:

$$\Delta sPRE = sPRE(protein + ligand) - sPRE(protein)$$

$$= \frac{S\,(protein\ +\ TEMPOL + ligand)}{S\,(proein\ + ligand +\ TEMPOL\ +\ Ascorbate)}$$

$$- \frac{S\,(protein +\ TEMPOL)}{S\,(protein + TEMPOL\ +\ Ascorbate)}$$

Where S (protein + TEMPOL+ ligand) is the signal amplitude of protein in the presence of ligand and TEMPOL; S (protein + ligand + TEMPOL + Ascorbate) is the diamagnetic reference of protein with ligand, and ascorbate is added to quench the activity of TEMPOL; S (protein + TEMPOL) is the signal amplitude of protein in the presence of TEMPOL; S (protein + TEMPOL + Ascorbate) is the diamagnetic reference of protein alone.

MD simulation

The structures of hLang and mLang were simulated using the protocol described by Blanco et al., with some modifications.[3] Briefly, the system was prepared with the leap module from the AMBER package using ff14SB and TIP3P force field for amino acid and water molecules.[4] The systems were first optimized using a conjugate gradient algorithm for 5000 steps, followed by 150 ps constant volume MD equilibration. The heating step was followed by a 300 ps of constant temperature and pressure simulation to equilibrate the system density. During these equilibration processes, the protein alpha-carbon atoms were constrained by a 5 kcal/mol/Å force constant using a harmonic potential. Next, a second equilibration MD of 10 ns was performed, in which the integration step was increased to 2 fs using the SHAKE algorithm, and the force constant for restrained alpha-carbons was decreased to 2 kcal/mol/Å followed by a 10 ns long MD simulation with no constraints. Finally, 1000 ns long production MD was carried out using the 'Hydrogen

Mass Repartition' method, which allows an integration step of 4 fs.[5] The trajectory processing and distance analysis were performed with the CPPTRAJ module of the AMBER package.[6]

General experimental details for compounds in Table 1

All thiazolopyrimidine chemicals were purchased from commercial suppliers: Activate Scientific, Sigma-Aldrich and Alfa Aesar and used as received unless otherwise specified. Compounds in **Table 1** that are not available from commercial suppliers are either synthesized according to procedures mentioned in the literature or here.[7] NMR spectra were recorded at either 295 K (300 MHz) or 300 K (600 MHz) at either Bruker AV 300 (300 MHz, 75 MHz) or Bruker AV 600 (600 MHz, 150 MHz) spectrometers. Chemical shifts are reported in ppm (δ) referenced to TMS (δ = 0.00 ppm), and $CHCl_3$ (7.26 ppm). Yields refer to isolated compounds, estimated to be > 95% pure as determined by NMR and LC-MS. TLC: Merck, TLC Silica gel 60 F254. LC/MS analysis was performed on an Agilent LC/MS 1260 analytical HPLC with DAD coupled to an Agilent 6120 single quadrupole mass spectrometer (ESI-SQ) equipped with a Thermo Fisher Scientific Accucore C18 column, 2.1 x 30 mm, 2.6 µm. Method: ESI+, flux: 0.8 ml/min, 5 - 95% CH_3CN in H_2O + 0.1% TFA, total runtime: 2.5 min. High-resolution mass spectra were recorded on an Agilent 6220A accurate-mass time-off flight mass spectrometer (ESI-TOF) with Agilent 1200 HPLC/DAD front-end.

Preparation of compound 8:

N-*Dodecyl*-5-oxo-5*H*-thiazolo[3,2-*a*] pyrimidine-6-carboxamide

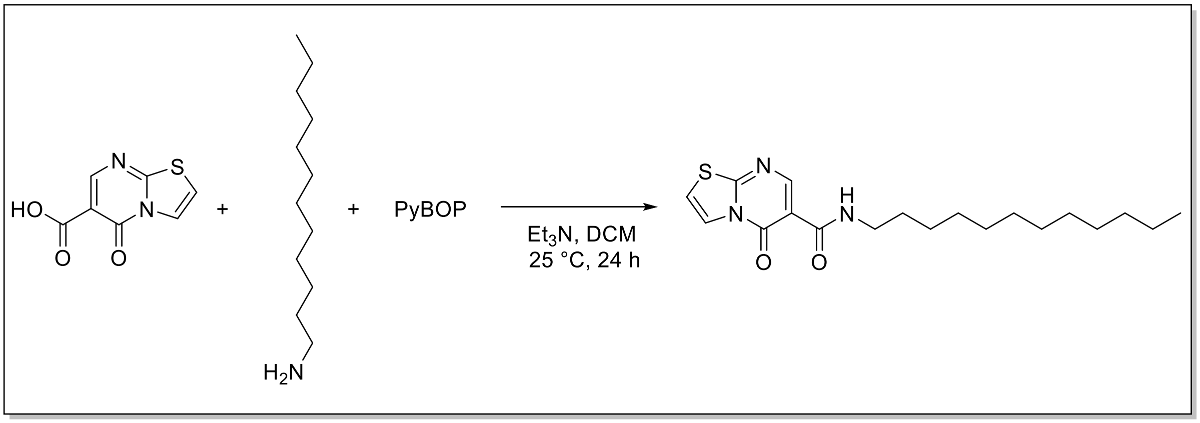

5-Oxo-5H-thiazolo[3,2-a] pyrimidine-6-carboxylic acid (98 mg, 0.5 mmol, 1.0 equiv) was placed in a pre-dried reaction tube equipped with screw cap and silicone septum. The tube was evacuated and flushed with N_2 three times and DCM (3.0 mL) was added. PyBOP (312 mg, 0.6 mmol, 1.2 equiv) and Et_3N (139 µL, 1.0 mmol, 2.0 equiv) were added to the reaction mixture. The reaction mixture was stirred at 25 °C for 5 mins. Dodecylamine (111 mg, 0.6 mmol, 1.2 equiv) dissolved in DCM (0.5 mL) was added to the reaction mixture. The reaction mixture was further stirred at 25 °C for 24 h. After completion, all volatiles were removed in vacuo and the residue was dissolved in DCM (50 mL). The organic layer was washed with 5% $KHSO_4$ aqueous solution (20 mL × 3). The organic layer was washed with 5% $NaHCO_3$ aqueous solution (20 mL × 3). The organic layer was washed with brine (50 mL). The organic phase was dried with Na_2SO_4, filtered and concentrated in vacuo. The residue was purified by an automated flash chromatography system using eluent (MeOH / DCM) to give compound 8 as an off-white solid (101 mg, 56% yield).

^{1}H NMR (600 MHz, $CDCl_3$) δ: 0.86 - 0.90 (m, 3 H), 1.22 - 1.42 (m, 18 H),1.63 (quin, J = 7.32 Hz, 2 H), 3.43 - 3.49 (m, 2 H), 7.22 (d, J = 4.88 Hz, 1 H), 8.14 (d, J = 4.88 Hz, 1 H), 8.80 (br s, 1 H), 9.10 (s, 1 H).

$^{13}C\{^{1}H\}$ NMR (150 MHz, $CDCl_3$) δ: 165.16, 163.16, 158.51, 157.81, 122.63, 113.76, 109.47, 39.64, 32.06, 29.79, 29.77, 29.74, 29.70, 29.69, 29.48, 29.47, 27.22, 22.82, 14.25.

HRMS (ESI+) m/z calc. for $C_{19}H_{30}N_3O_2S$: 364.2053, found 364.2061 $[M+H]^+$.

NMR of N-*dodecyl*-5-oxo-5*H*-thiazolo[3,2-*a*] pyrimidine-6-carboxamide

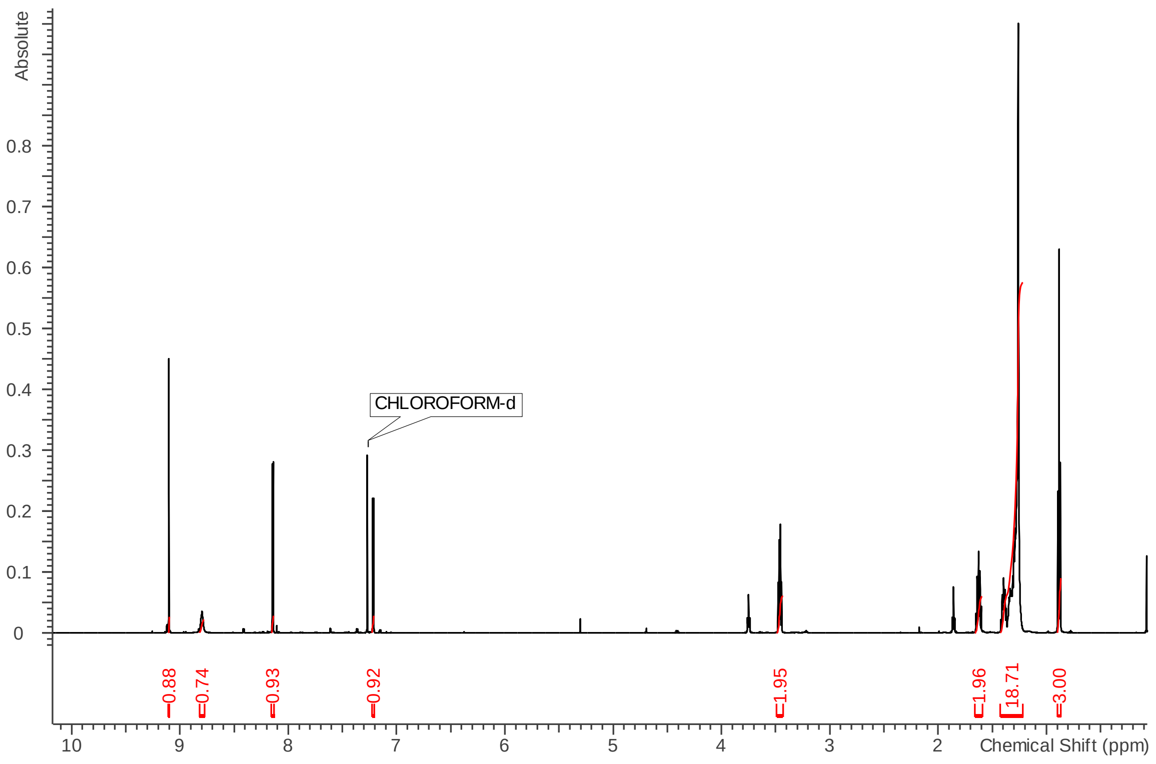

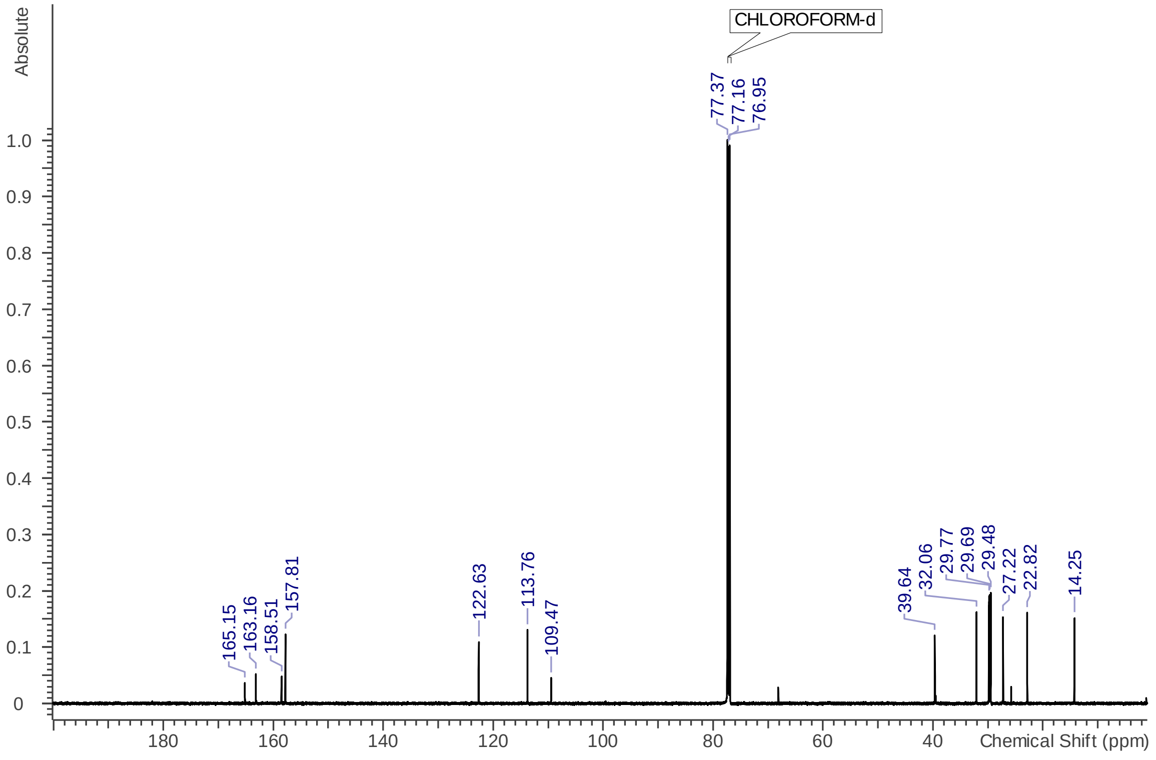

Preparation of compound 2:

***N*-(2-Methoxybenzyl)-5-oxo-5*H*-thiazolo[3,2-*a*] pyrimidine-6-carboxamide**

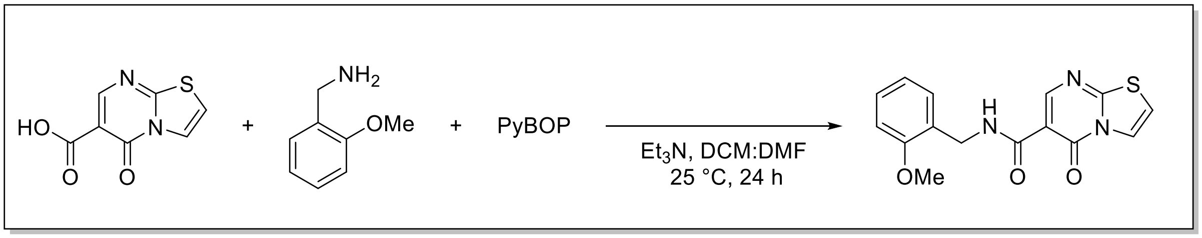

5-Oxo-5H-thiazolo[3,2-a] pyrimidine-6-carboxylic acid (98 mg, 0.5 mmol, 1.0 equiv) was placed in a pre-dried reaction tube equipped with screw cap and silicone septum. The tube was evacuated and flushed with N_2 three times. DCM (3.0 mL) and DMF (1.0 mL) was added to the reaction tube. PyBOP (312 mg, 0.6 mmol, 1.2 equiv) and Et_3N (139 µL, 1.0 mmol, 2.0 equiv) were added to the reaction mixture. The reaction mixture was stirred at 25 °C for 5 mins. 2-Methoxybenzylamine (82 mg, 0.6 mmol, 1.2 equiv) dissolved in DCM (0.5 mL) was added to the reaction mixture. The reaction mixture was further stirred at 25 °C for 24 h. After completion, all volatiles were removed in vacuo and the residue was dissolved in DCM (50 mL). The organic layer was washed with 5% $KHSO_4$ aqueous solution (20 mL × 3). The organic layer was washed with 5% $NaHCO_3$ aqueous solution (20 mL × 3). The organic layer was washed with brine (50 mL). The organic phase was dried with Na_2SO_4, filtered, and concentrated in vacuo. The residue purified by automated flash chromatography system using eluent (MeOH / DCM) to give compound 2 as an off white solid (100 mg, 63% yield).

^{1}H NMR (300 MHz, $CDCl_3$) δ: 3.90 (s, 3 H), 4.66 (d, J = 5.80 Hz, 2 H), 6.85 - 6.97 (m, 2 H), 7.18 (d, J = 4.88 Hz, 1 H) 7.21 - 7.29 (m, 1 H) 7.34 (dd, J = 7.32, 1.53 Hz, 1 H) 8.12 (d, J = 4.88 Hz, 1 H) 9.10 (s, 1 H) 9.24 (br s, 1 H)

$^{13}C\{^{1}H\}$ NMR (75 MHz, CDCl3) δ: 165.21, 163.07, 158.33, 157.88, 157.71, 129.50, 128.76, 126.61, 122.66, 120.69, 113.67, 110.44, 109.48, 55.53, 39.19.

HRMS (ESI+) m/z calc. for $C_{15}H_{14}N_3O_3S$: 316.0750, found 316.0718 $[M+H]^+$.

NMR of *N*-(2-methoxybenzyl)-5-oxo-5*H*-thiazolo[3,2-*a*] pyrimidine-6-carboxamide

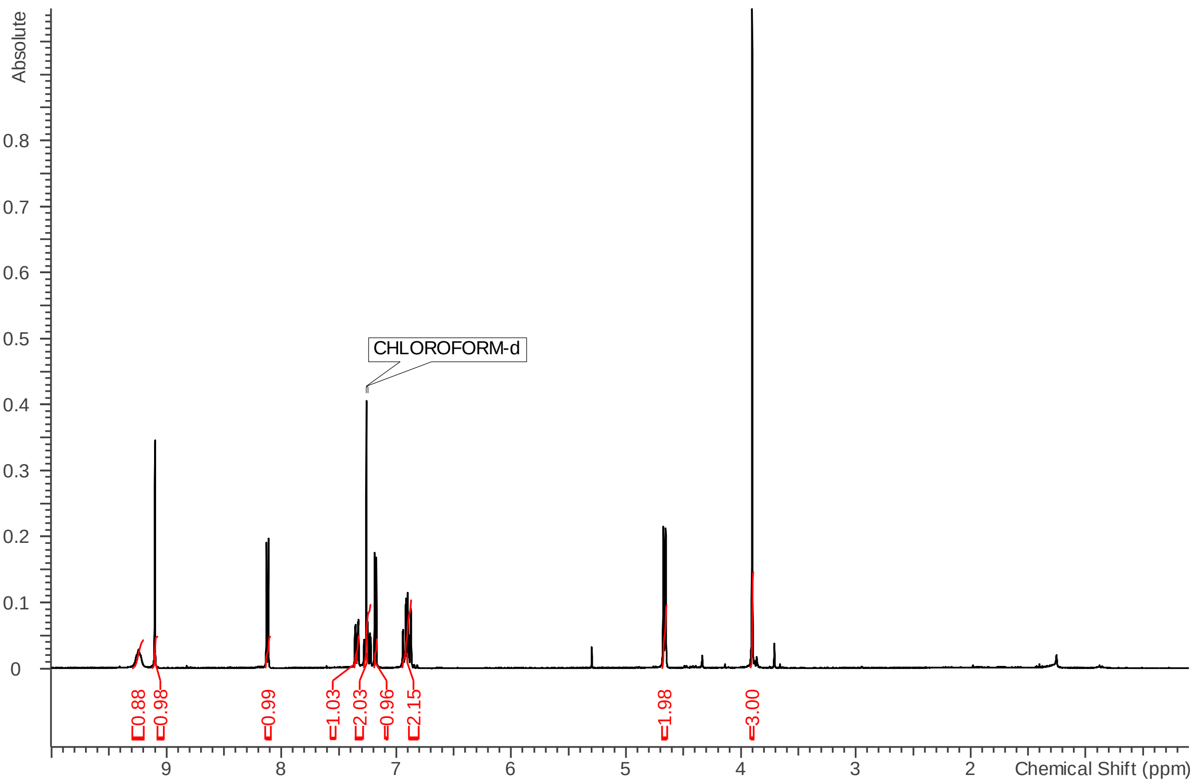

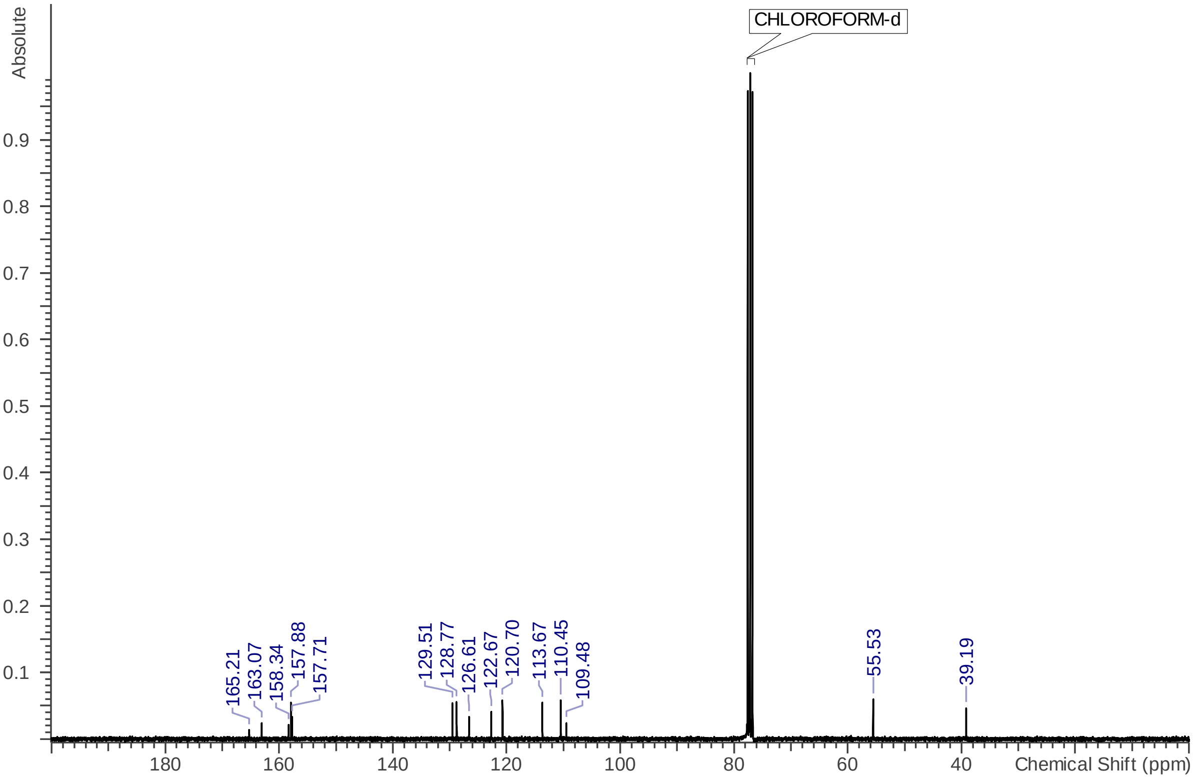

General experimental details for compounds in Table S3

All peptides were synthesized by microwave assisted SPPS on a Liberty Blue synthesizer, using the Fmoc strategy on preloaded Wang resin (0.05 mmol) and Oxyma Pure and DIC as coupling agents.

Peptides were cleaved from the resin, as well as sensitive sidechain protecting groups, using 3 mL of TFA/DODT/TIS/H2O (90:5:2.5:2.5) for 1 h at r.t. Peptides were then precipitated with cold diethyl ether (20 mL) and centrifuged for 5 min at 900 x g. The supernatant solution was discarded, and this process was repeated 2 times. Peptides were dried and redissolved in water to be purified by reverse phase HPLC on an ACE-C18 column (5 um, 250 x 21.2 mm) with a flow rate of 20 mL/min. UV detection was performed at 212 nm.

Analytical HPLC gradient 90% → 70% solvent A in 15 min at 1 mL/min for all peptides. Solvent A: H_2O 0.1% TFA and solvent B: ACN.

AWS

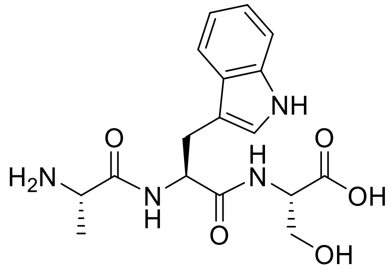

HRMS (m/z): $[M+H]^+$ calc. for $C_{17}H_{22}N_4O_5$, 363.1663; found 363.1693.

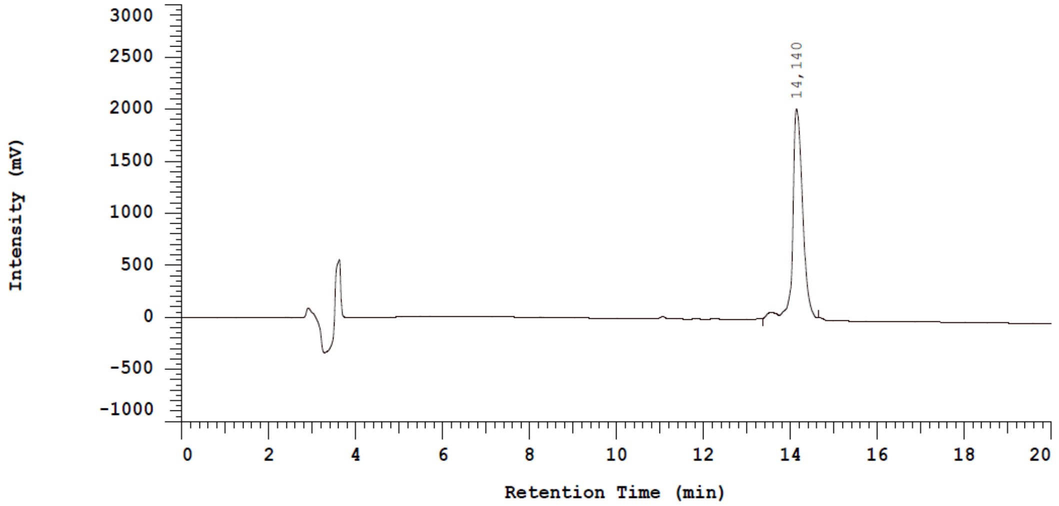

HPLC Rt = 14.1 min (Grad: water+0.1% TFA/acetonitrile (90:20) → (70:30), 15 min, 1 mL/min, λ = 212 nm).

^{1}H NMR (500 MHz, D_2O) δ: 7.62 – 7.59 (m, 1H, arom Trp), 7.48 – 7.45 (m, 1H, arom Trp), 7.25 (s, 1H, pyrrole), 7.22 – 7.18 (m, 1H, arom Trp), 7.14 – 7.10 (m, 1H, arom Trp), 4.68 (t, J = 7.6 Hz, 1H Hα-Ser), 4.32 (t, J = 4.3 Hz, 1H, Hα-Trp), 4.01 (q, J = 7.1 Hz, 1H, Hα-Ala), 3.80 (dd, J = 11.7, 4.5 Hz, 1H, Hβ-Trp), 3.71 (dd, J = 11.8, 4.2 Hz, 1H, Hβ-Trp), 3.26 (dd, J = 7.6, 2.7 Hz, 2H, Hβ-Ser), 1.47 (d, J = 7.1 Hz, 3H, Ala-CH_3).

^{13}C NMR (126 MHz, D_2O) δ: 172.98, 172.49, 170.45 (3 x C=O), 136.08, 126.72, 124.50, 121.87, 119.22, 118.09, 111.84, 108.53 (arom), 61.16 (Cβ-Trp), 54.81, 54.69 (Cα-Trp, Cα-Ser), 48.78 (Cα-Ala), 26.93 (Cβ-Ser), 16.41 (Cβ-Ala).

^{1}H NMR 500 MHz in D_2O measured at 298K

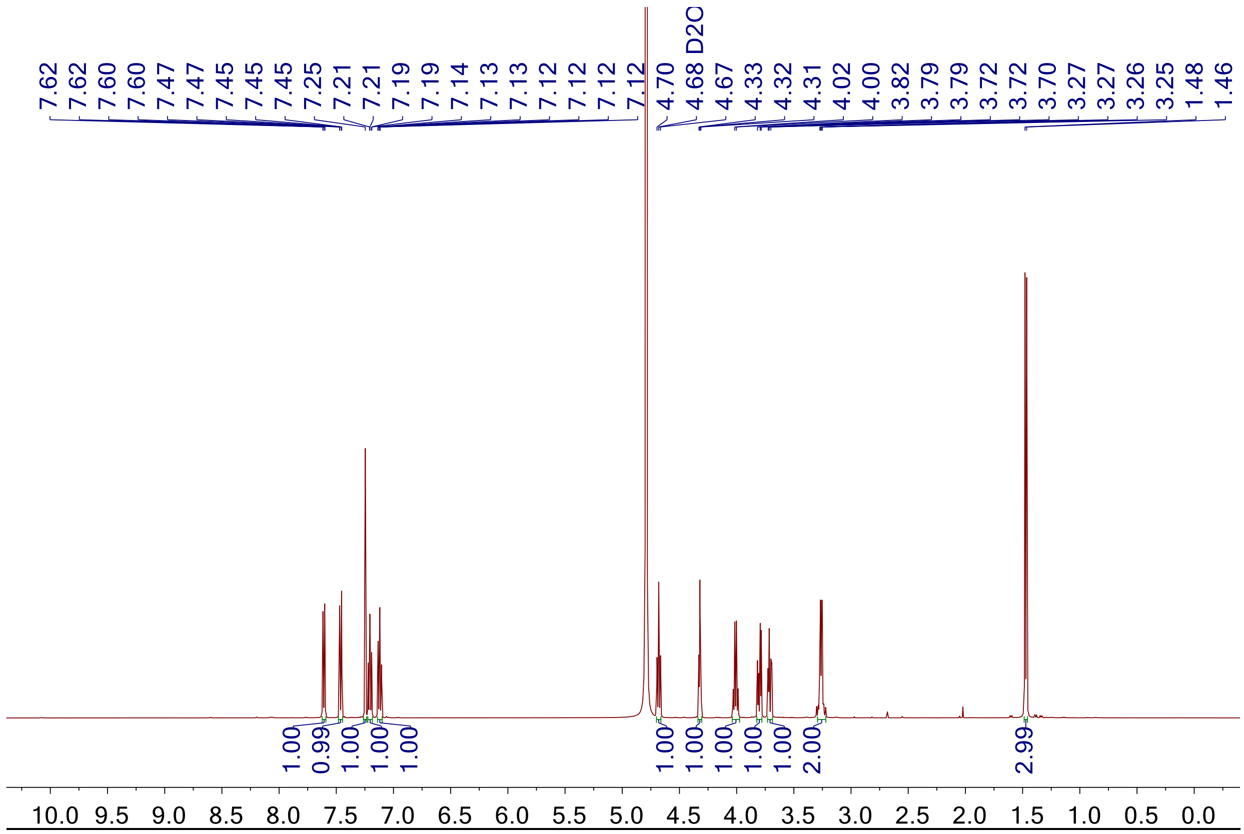

^{13}C NMR 500 MHz in D_2O measured at 298K

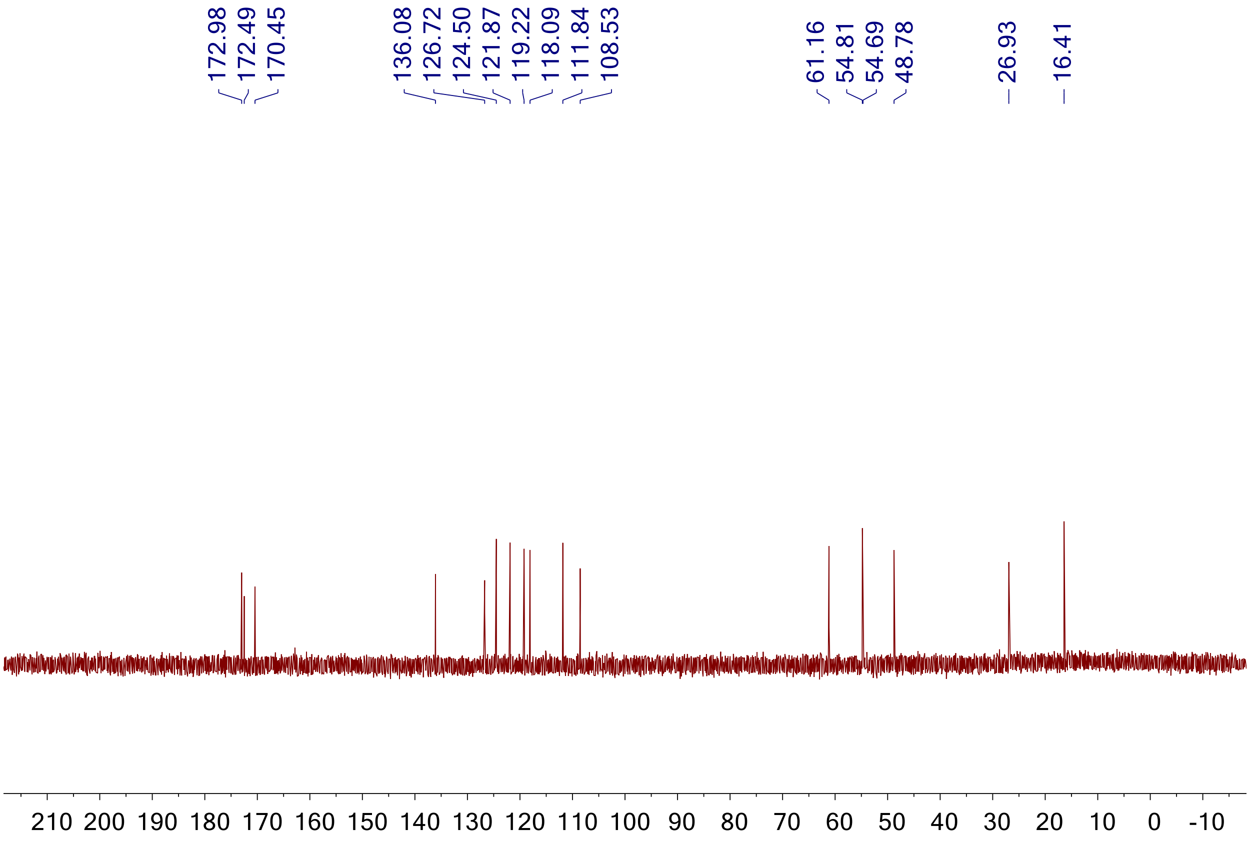

SAWS

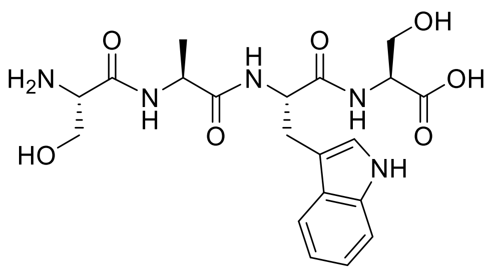

HRMS (m/z): $[M+H]^+$ calc. for $C_{20}H_{27}N_5O_7$, 450.1983; found 450.2024.

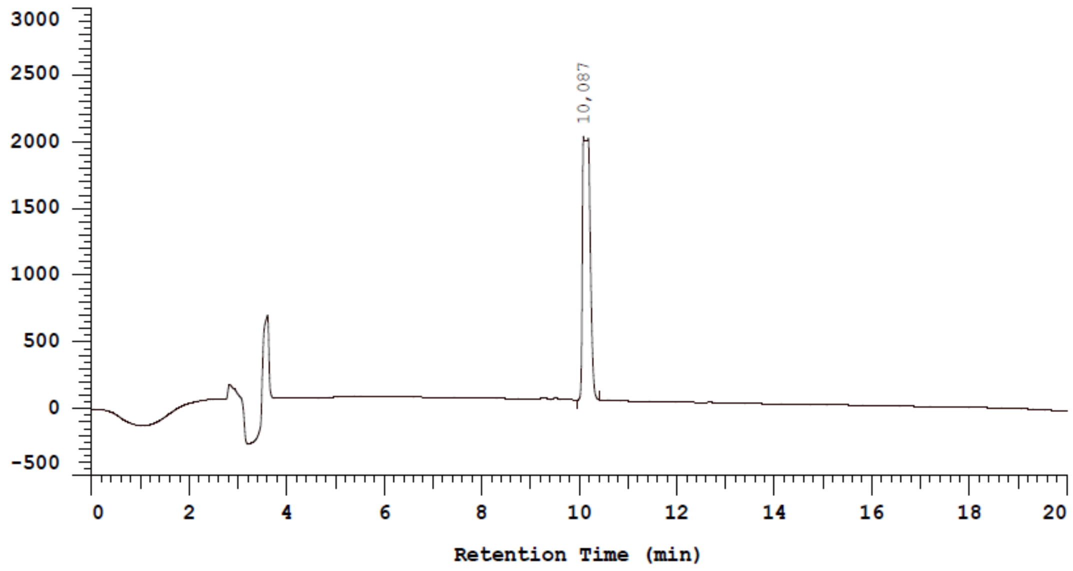

HPLC Rt = 10.1 min (Grad: water+0.1% TFA/acetonitrile (90:20) → (70:30), 15 min, 1 mL/min, λ = 212 nm).

^{1}H NMR (500 MHz,D_2O) δ|: 7.58 – 7.56 (m, 1H, arom Trp), 7.45 – 7.43 (m, 1H, arom Trp), 7.20 – 7.17 (m, 2H, pyrrol, arom Trp), 7.12 – 7.10 (m, 1H, arom Trp), 4.64 (t, *J* = 7.2 Hz, 1H, Hα-Ser-Cterm), 4.34 (t, *J* = 4.4 Hz, 1H, Hα-Trp), 4.27 (q, *J* = 7.2 Hz, 1H, Hα-Ala), 4.05 – 4.03 (m, 1H, Hα-Ser-Nterm), 3.83 – 3.77 (m, 2H, Hβ-Trp), 3.73 – 3.67 (m, 2H, Hβ-Ser-Nterm), 3.23 (qd, *J* = 14.6, 7.2 Hz, 2H, Hβ-Ser-Nterm), 1.24 (d, *J* = 7.2 Hz, 3H, Ala-CH_3).

^{13}C NMR (126 MHz, D_2O) δ: 174.14, 173.06, 172.51, 167.60 (4 x C=O), 136.07, 126.85, 124.47, 121.83, 119.25, 118.08, 111.80, 108.44 (arom), 61.07, 60.01 (Cβ-Trp, Cβ-Ser-Nterm), 54.63, 54.34, 54.32 (Cα-Trp, Cα-Ser, Cα-Ser), 49.84 (Cα-Ala), 26.83 (Cβ-Ser-Cterm), 16.21 (Cβ-Ala).

^{1}H NMR 500 MHz in D_2O measured at 298K

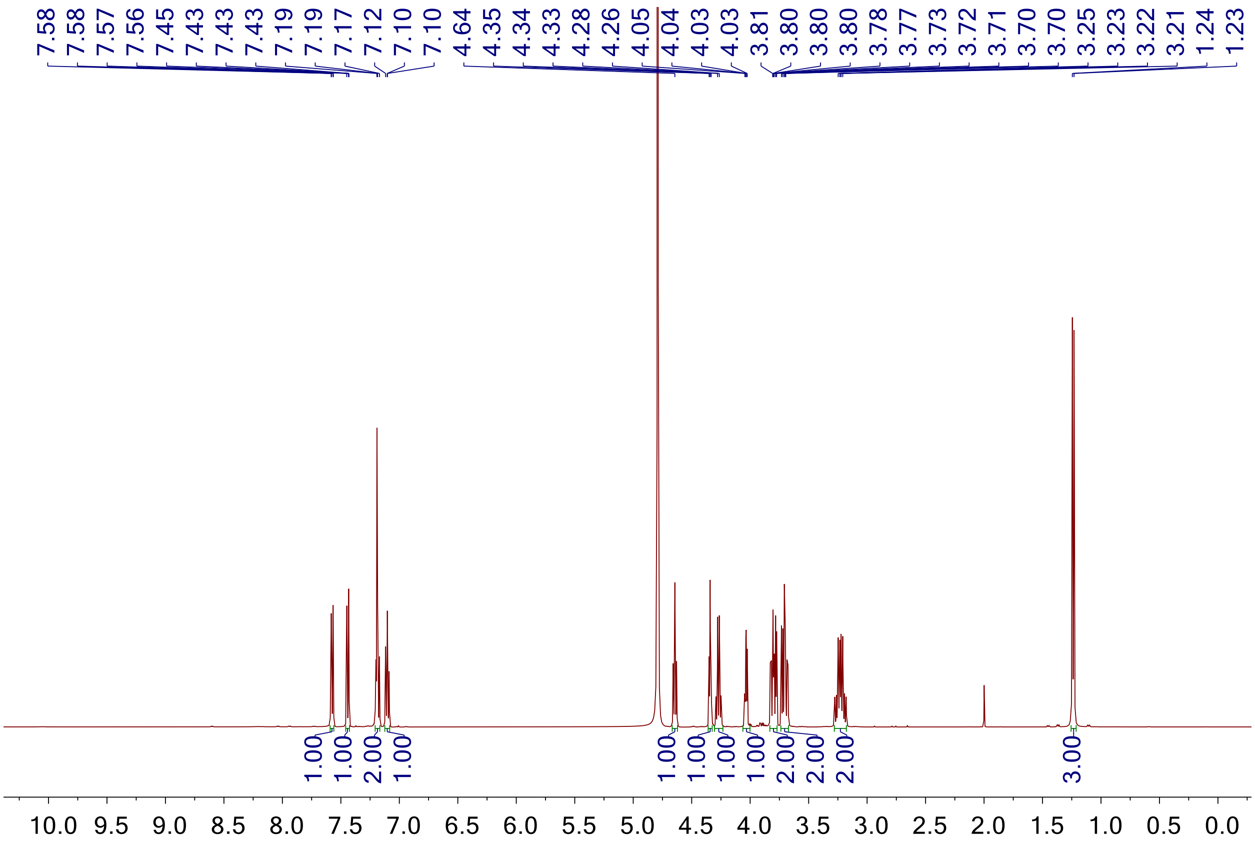

^{13}C NMR 500 MHz in D_2O measured at 298K

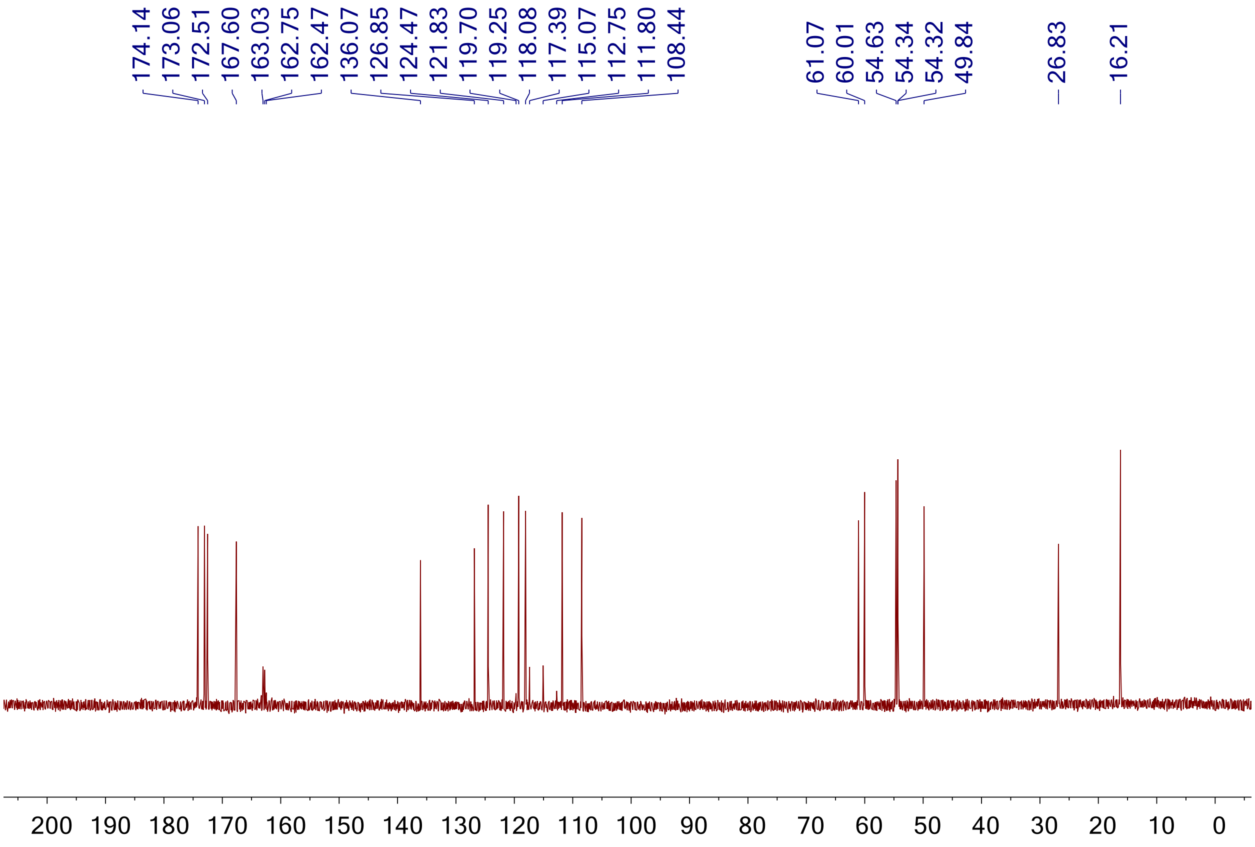

EPSAWS

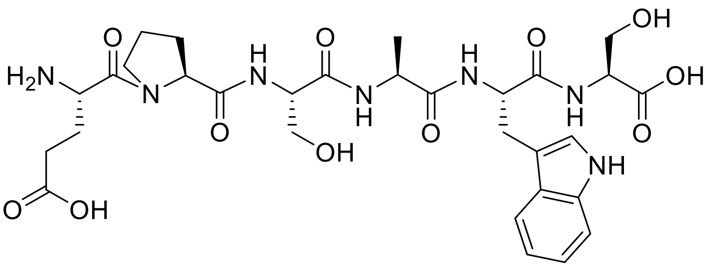

HRMS (m/z): $[M+H]^+$ calc. for $C_{30}H_{41}N_7O_{11}$, 676.2937; found 676.2987.

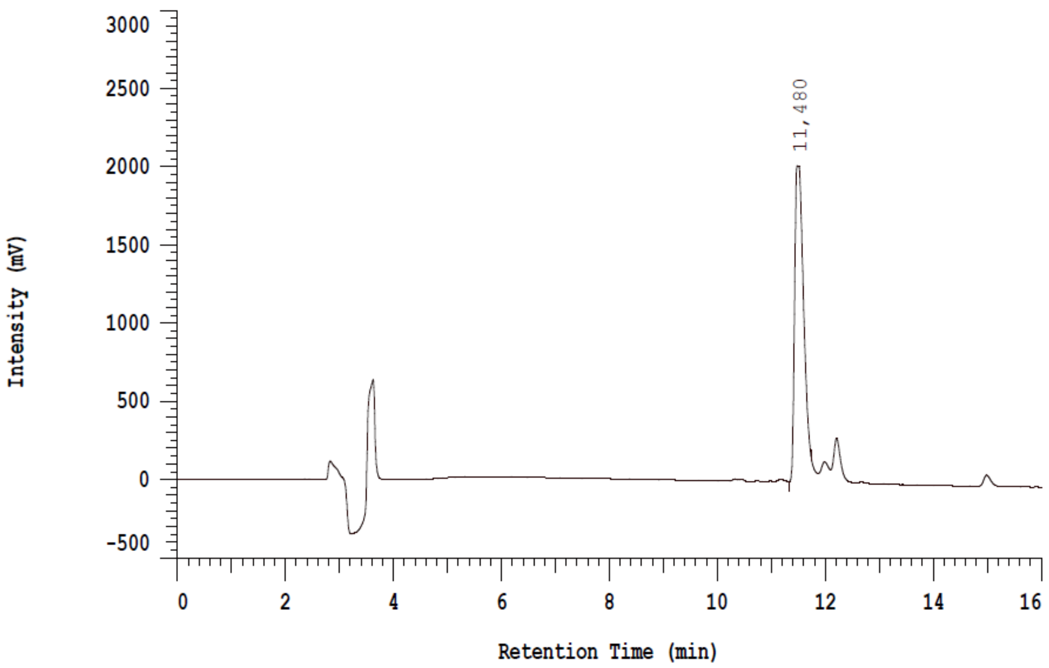

HPLC Rt = 11.5 min (Grad: water+0.1% TFA/acetonitrile (90:20) → (70:30), 15 min, 1 mL/min, λ = 212 nm).

^{1}H NMR (500 MHz, D_2O) δ: 7.57 (d, J = 7.9, 1.1 Hz, 1H, arom Trp), 7.44 (d, J = 8.3, 1.1 Hz, 1H, arom Trp), 7.21 – 7.14 (m, 2H, pyrrol, arom Trp), 7.13 – 7.04 (m, 1H, arom Trp), 4.64 (t, J = 7.2 Hz, 1H, Hα-Ser-Cterm), 4.49 – 4.41 (m, 1H, Hα-Pro), 4.38 – 4.32 (m, 2H, Hα-Trp, Ha-Glu), 4.29 (t, J = 5.6 Hz, 1H, Hα-Ser), 4.23 (q, J = 7.2 Hz, 1H, Hα-Ala), 3.86 – 3.78 (m, 1H, Hβ-Trp), 3.75 – 3.61 (m, 4H, Hβ-Trp, 2Hβ-Ser, Hδ-Pro), 3.52 – 3.44 (m, 1H, Hδ-Pro), 3.31 – 3.14 (m, 2H, Hβ-Ser-Cterm), 2.56 – 2.45 (m, 2H, Hγ-Glu), 2.33 – 2.22 (m, 1H, Hβ-Pro), 2.20 – 2.03 (m, 2H, Hβ-Glu), 1.97 – 1.88 (m, 2H, Hγ-Pro), 1.87 – 1.74 (m, 1H, Hβ-Pro), 1.23 (d, J = 7.2 Hz, 3H, Ala-CH_3).

^{13}C NMR (126 MHz, D_2O) δ: 176.03, 175.76, 174.28, 173.65, 173.08, 172.55, 171.58, 167.78, 163.06, 162.78 (7 x C=O), , 136.07, 126.84, 124.24, 121.84, 119.21, 118.09, 117.40, 115.08, 111.77, 108.65 (arom), 61.11, 60.84, 60.39 (Cβ-Trp, Cβ-Ser, Cβ-Pro), 55.57 (Cα-Ser), 54.65 (Cα-

Trp), 54.26 (Cα-Ser-Cterm), 50.85 (Cα-Glu), 49.75 (Cα-Ala), 47.91 (Cδ-Pro), 29.32 (Cβ-Pro), 28.44 (Cγ-Glu), 26.88 (Cβ-Ser-Cterm), 24.74, 24.64, 21.75 (Cβ-Glu, Cγ-Pro), 16.22 (Cβ-Ala).

<u>^{1}H NMR 500 MHz in D_2O measured at 298K</u>

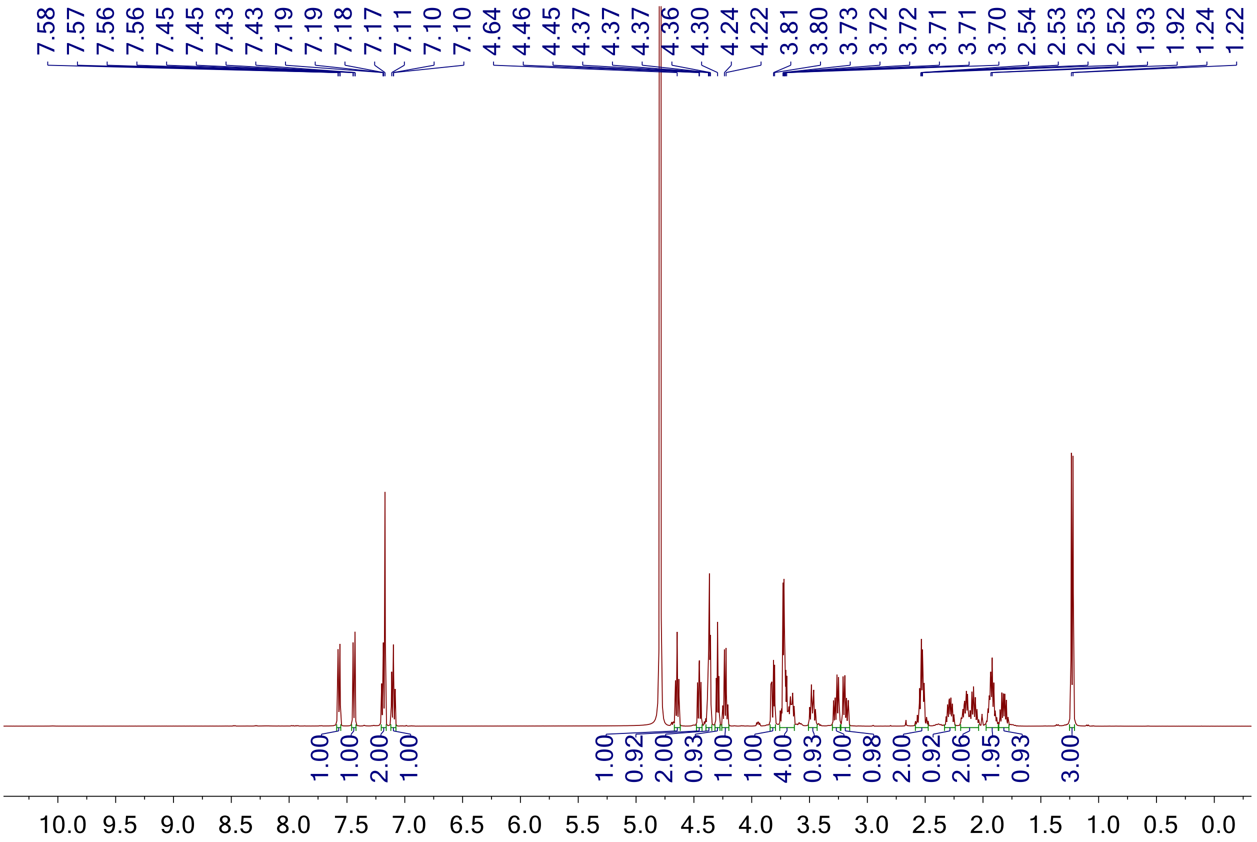

^{13}C NMR 500 MHz in D_2O measured at 298K

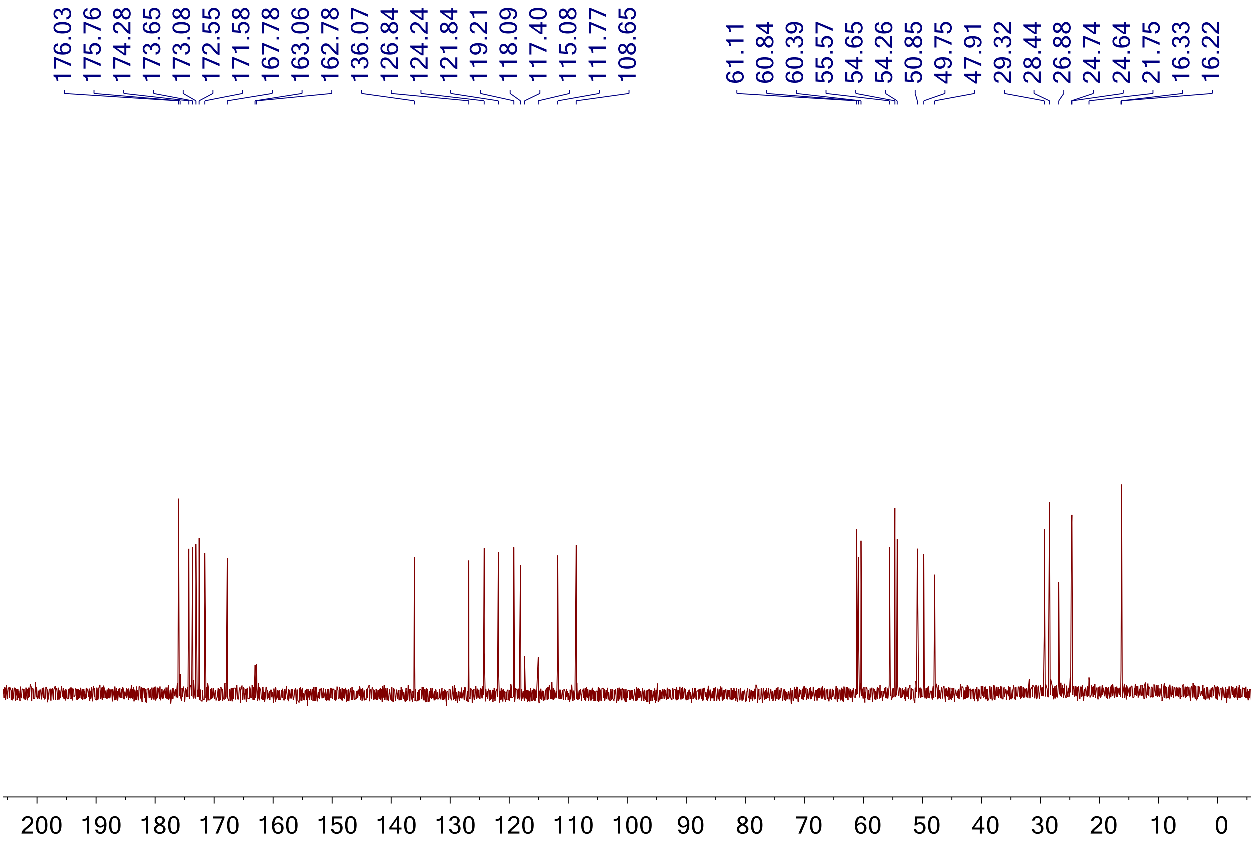

SWS

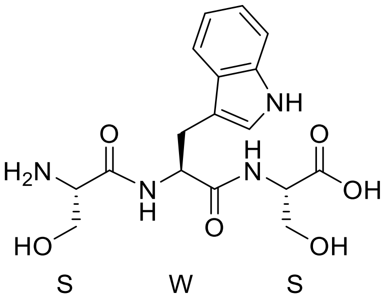

HRMS (m/z): $[M+H]^+$ calc. for $C_{17}H_{22}N_4O_6$, 379.1612; found 379.1640.

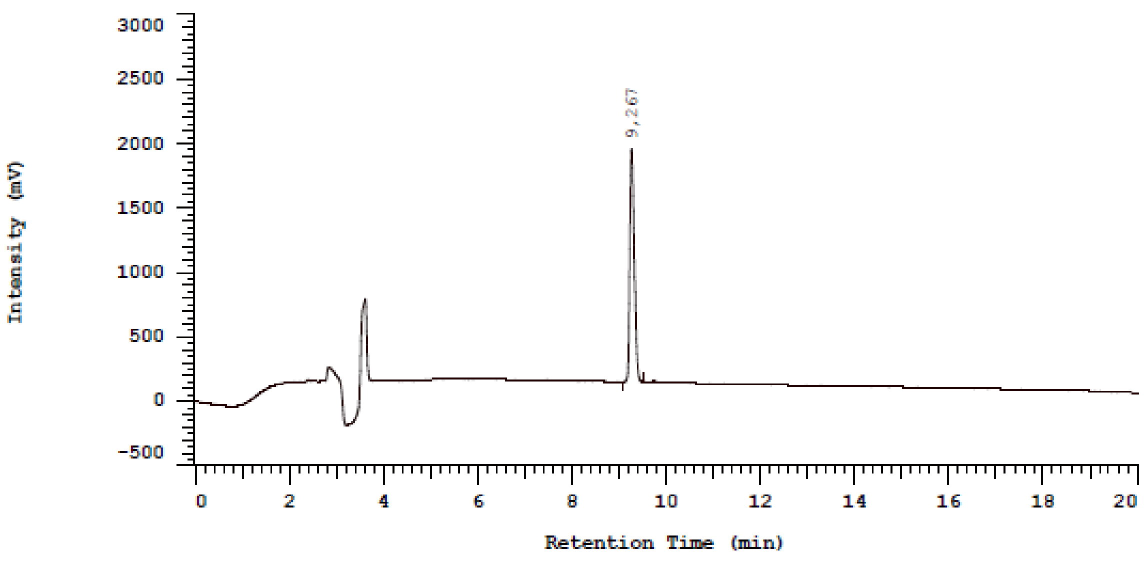

HPLC Rt = 9.3 min (Grad: water+0.1% TFA/acetonitrile (90:20) → (70:30), 15 min, 1 mL/min, λ = 212 nm).

^{1}H NMR (500 MHz, D_2O) δ: 7.61 (d, *J* = 8.0 Hz, 1H, arom Trp), 7.46 (d, *J* = 8.1 Hz, 1H, arom Trp), 7.25 (s, 1H), 7.24 – 7.17 (m, 1H, pyrrol), 7.12 (t, *J* = 8.0 Hz, 1H, arom Trp), 4.74 (t, *J* = 7.4 Hz, 1H, Hα-Ser-Cterm), 4.34 (t, *J* = 4.3 Hz, 1H, Hα-Trp), 4.09 – 4.03 (m, 1H, Hα-Ser-Nterm), 3.91 (qd, *J* = 12.3, 5.1 Hz, 2H, Hβ-Ser-Nterm), 3.80 (dd, *J* = 11.8, 4.6 Hz, 1H, Hβ-Trp), 3.71 (dd, *J* = 11.8, 4.2 Hz, 1H, Hβ-Trp), 3.32 – 3.22 (m, 2H, Hβ-Ser-Cterm)).

^{13}C NMR (126 MHz, D_2O) δ: 172.88, 172.51, 167.38 (3 x C=O), 136.07, 126.75, 124.52, 121.87, 119.22, 118.10, 111.83, 108.50 (arom), 61.12 (Cβ-Trp), 60.01 (Cβ-Ser), 54.77, 54.69 (Cα-Trp, Cα-Ser-Cter), 54.31 (Cα-Ser-Nter), 27.06 (Cβ-Ser-Cter).

^{1}H NMR 500 MHz in D_2O measured at 298K

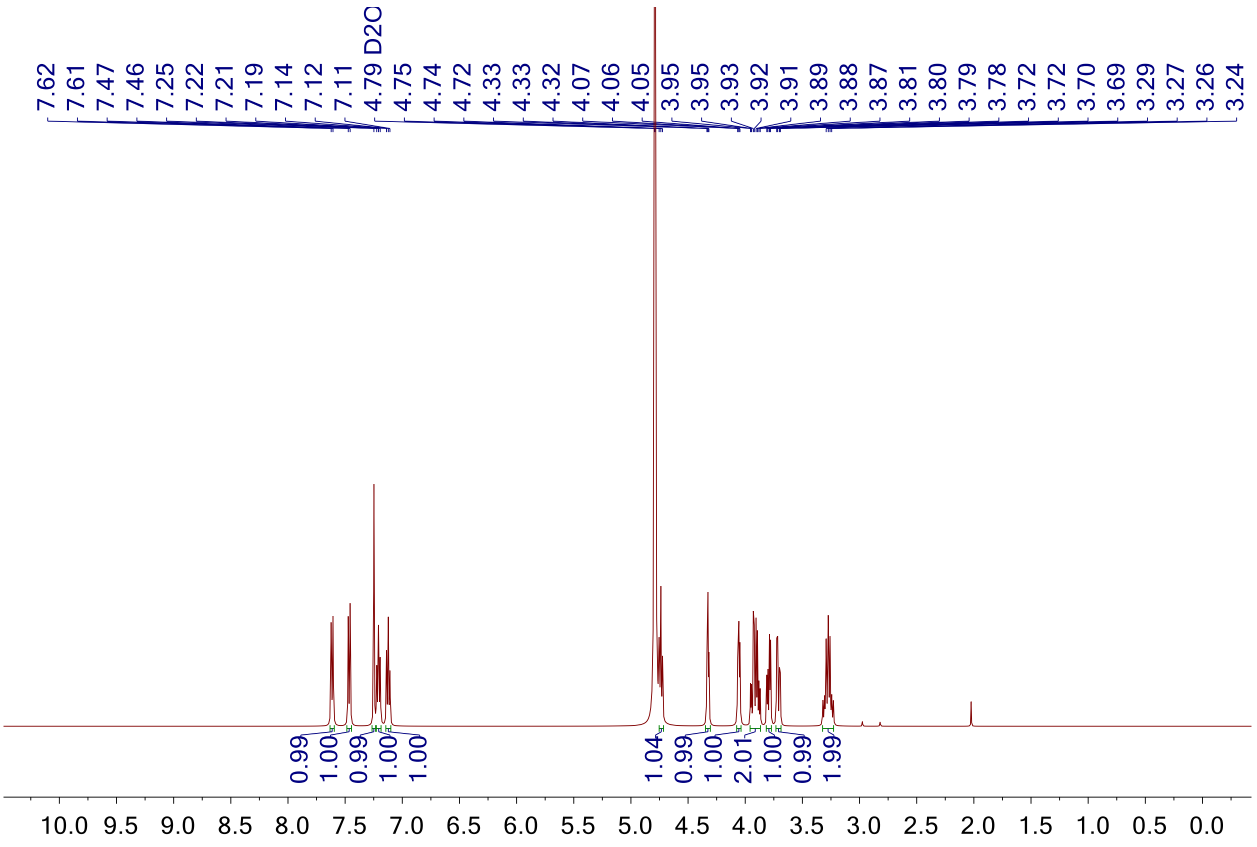

^{13}C NMR 500 MHz in D_2O measured at 298K

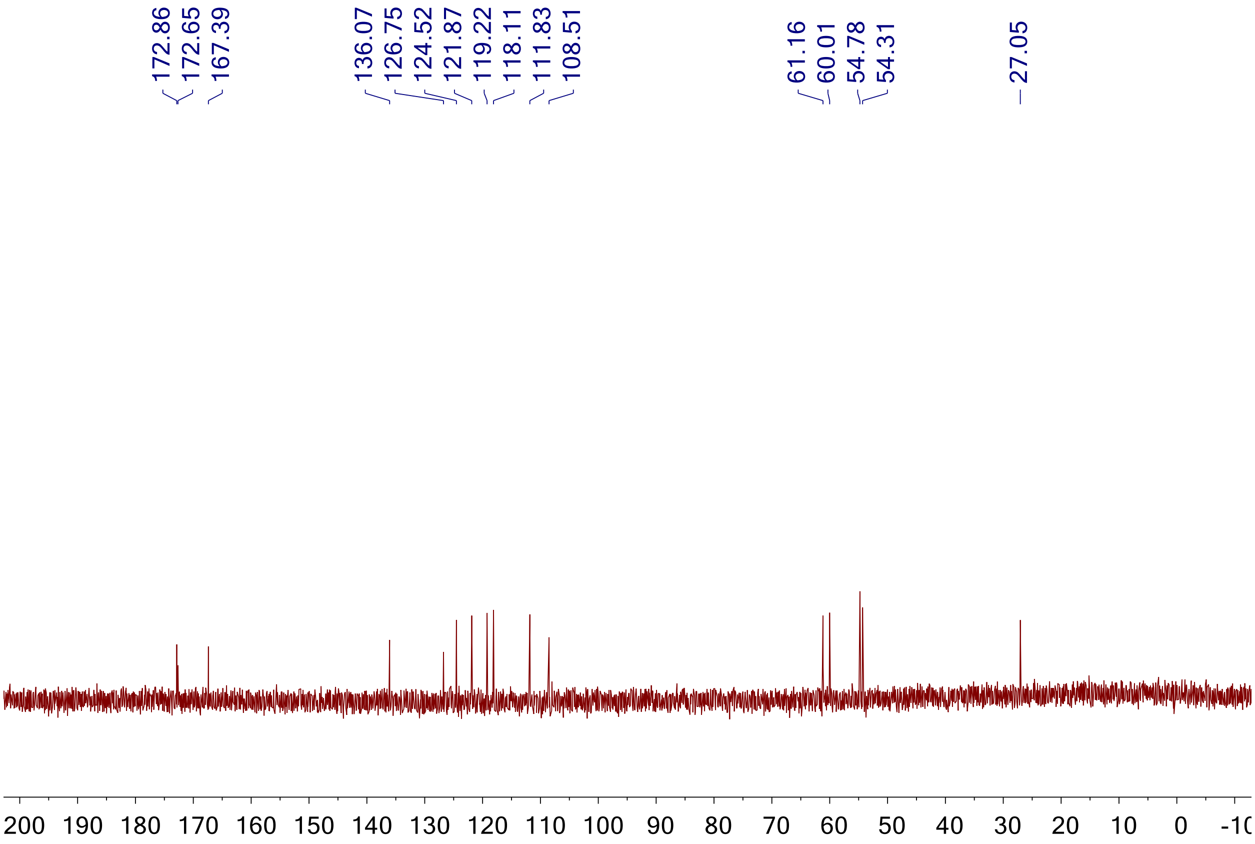

SWS

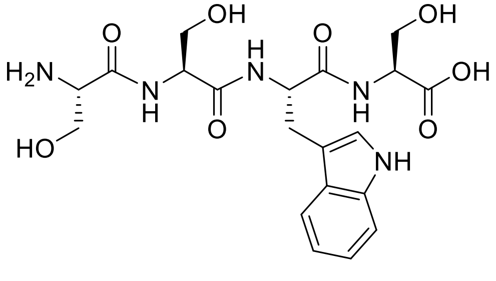

HRMS (m/z): $[M+H]^+$ calc. for $C_{20}H_{27}N_5O_8$, 466.1932; found 466.1973.

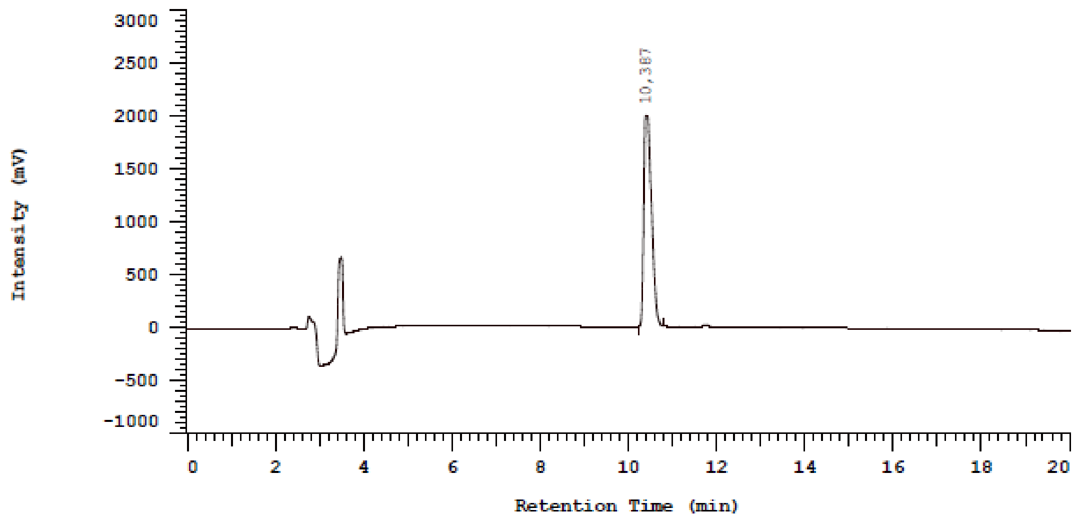

HPLC Rt = 10.4 min (Grad: water+0.1% TFA/acetonitrile (90:20) → (70:30), 15 min, 1 mL/min, λ = 212 nm).

^{1}H NMR (500 MHz, D_2O) δ: 7.60 (d, *J* = 7.9 Hz, 1H, arom Trp), 7.46 (d, *J* = 8.2 Hz, 1H, arom Trp), 7.24 – 7.17 (m, 2H, arom Trp, pyrrol), 7.12 (t, *J* = 7.5 Hz, 1H, arom Trp), 4.72 (t, *J* = 6.9 Hz, 1H, Hα-Ser-Cterm), 4.44 (t, *J* = 5.4 Hz, 1H, Hα-Ser), 4.34 (t, *J* = 4.3 Hz, 1H, Hα-Trp), 4.14 – 4.06 (m, 1H, Hα-Ser´), 3.91 – 3.67 (m, 6H, 2Hβ-Trp, Hβ-Ser, Hβ-Ser´), 3.26 (qd, *J* = 14.6, 7.0 Hz, 2H, Hβ-Ser-Cterm).

^{13}C NMR (126 MHz, D_2O) δ: 172.94, 172.94, 170.82, 167.94 (4 x C=O), 136.07, 126.84, 124.53, 121.86, 119.25, 118.15, 111.80, 108.42 (arom), 61.16, 60.77, 59.96 (Cβ-Trp, Cβ-Ser, Cβ-Ser´), 55.58, 54.97, 54.39, 54.31 (Cα-Trp, Cα-Ser, Cα-Ser´, Cα-Ser-Cterm), 26.93 (Cβ-Ser-Cterm).

^{1}H NMR 500 MHz in D_2O measured at 298K

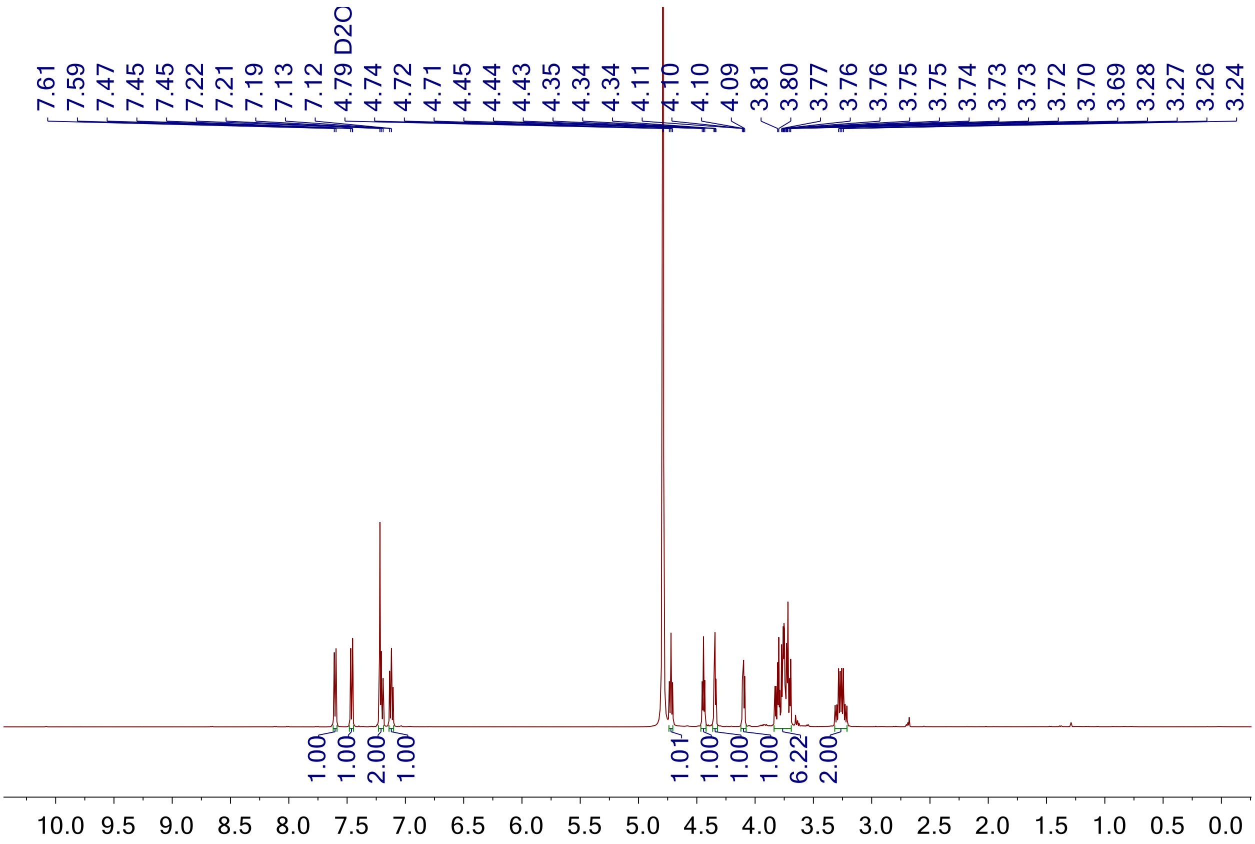

^{13}C NMR 500 MHz in D_2O measured at 298K

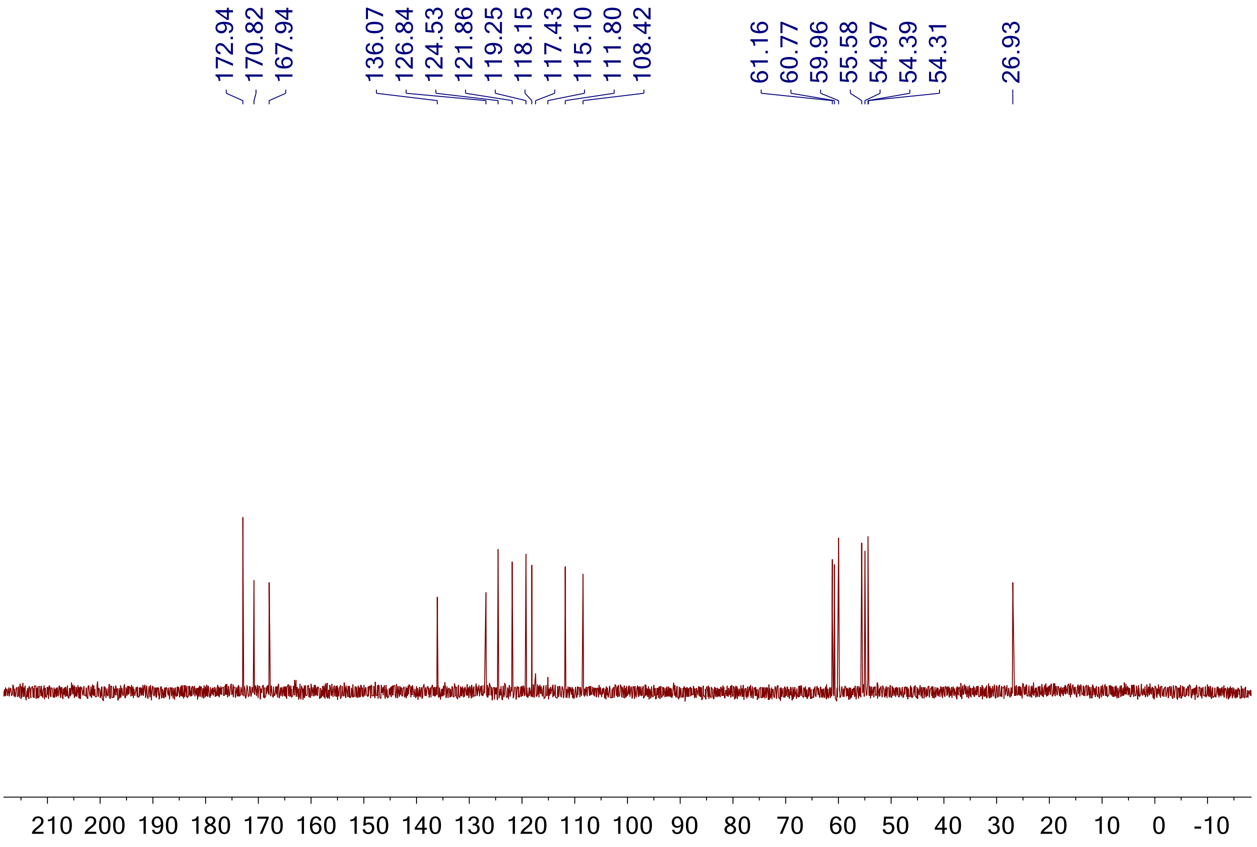

EPSWS

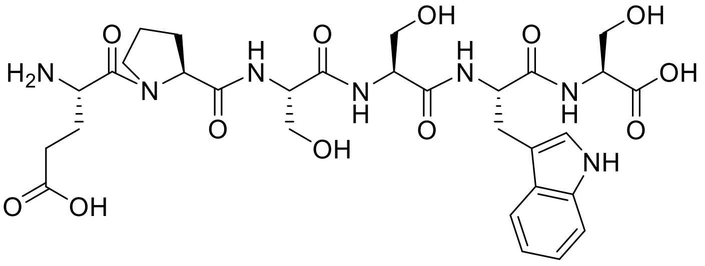

HRMS (m/z): $[M+H]^+$ calc. for $C_{30}H_{41}N_7O_{12}$, 492.2886; found 492.2947.

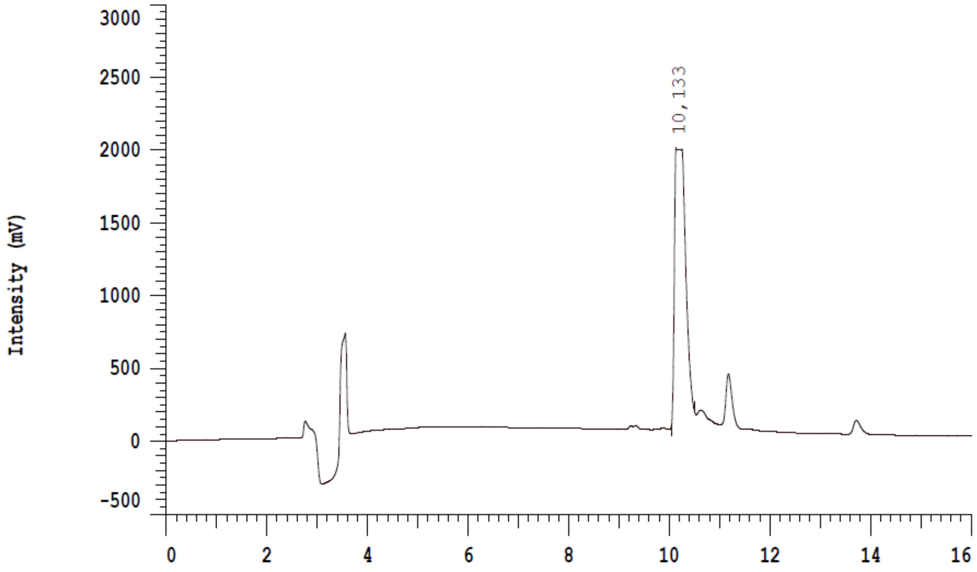

HPLC Rt = 10.1 min (Grad: water+0.1% TFA/acetonitrile (90:20) → (70:30), 15 min, 1 mL/min, λ = 212 nm).

^{1}H NMR (500 MHz, D_2O) δ: 7.59 (d, J = 7.9 Hz, 1H, arom Trp), 7.46 (d, J = 8.7 Hz, 1H, arom Trp), 7.25 – 7.18 (m, 2H, pyrrol, arom Trp), 7.16 – 7.09 (m, 1H, arom Trp), 4.71 (t, J = 7.1 Hz, 1H, Hα-Ser-Cterm), 4.49 (t, J = 7.4 Hz, 1H, Hα-Pro), 4.43 – 4.32 (m, 4H, Hα-Trp, Hα-Glu, Hα-Ser, Hα-Ser), 3.88 – 3.71 (m, 6H, 2Hβ-Trp, 2Hβ-Ser, 2Hβ-Ser), 3.70 – 3.62 (m, 1H, Hδ-Pro), 3.48 – 3.40 (m, 1H, Hδ-Pro), 3.31 (dd, J = 14.8, 6.6 Hz, 1H, Hβ-Ser-Cterm), 3.22 (dd, J = 14.8, 6.6 Hz, 1H, Hβ-Ser-Cterm), 2.61 – 2.44 (m, 2H, Hγ-Glu), 2.38 – 2.27 (m, 1H, Hβ-Pro), 2.20 – 2.02 (m, 2H, Hβ-Glu), 1.99 – 1.90 (m, 2H, Hγ-Pro), 1.90 – 1.80 (m, 1H, Hβ-Pro).

^{13}C NMR (126 MHz, D_2O) δ: 175.99, 173.81, 173.03, 172.61, 171.88, 171.14, 167.81 (7 x C=O), 136.09, 126.89, 124.28, 121.87, 119.23, 118.13, 111.80, 108.69 (arom), 61.13, 60.78, 60.76, 60.42 (Cβ-Trp, Cβ-Ser, Cβ-Ser, Cβ-Pro), 55.86, 55.52, 54.70, 54.42 (Cα-Trp, Cα-Ser, Cα-Ser, Cα-Ser), 50.89 (Cα-Glu), 47.89 (Cδ-Pro), 29.38 (Cβ-Pro), 28.42 (Cγ-Glu), 26.99 (Cβ-Ser-Cterm), 24.75, 24.68 (Cβ-Glu, Cγ-Pro).

^{1}H NMR 500 MHz in D_2O measured at 298K

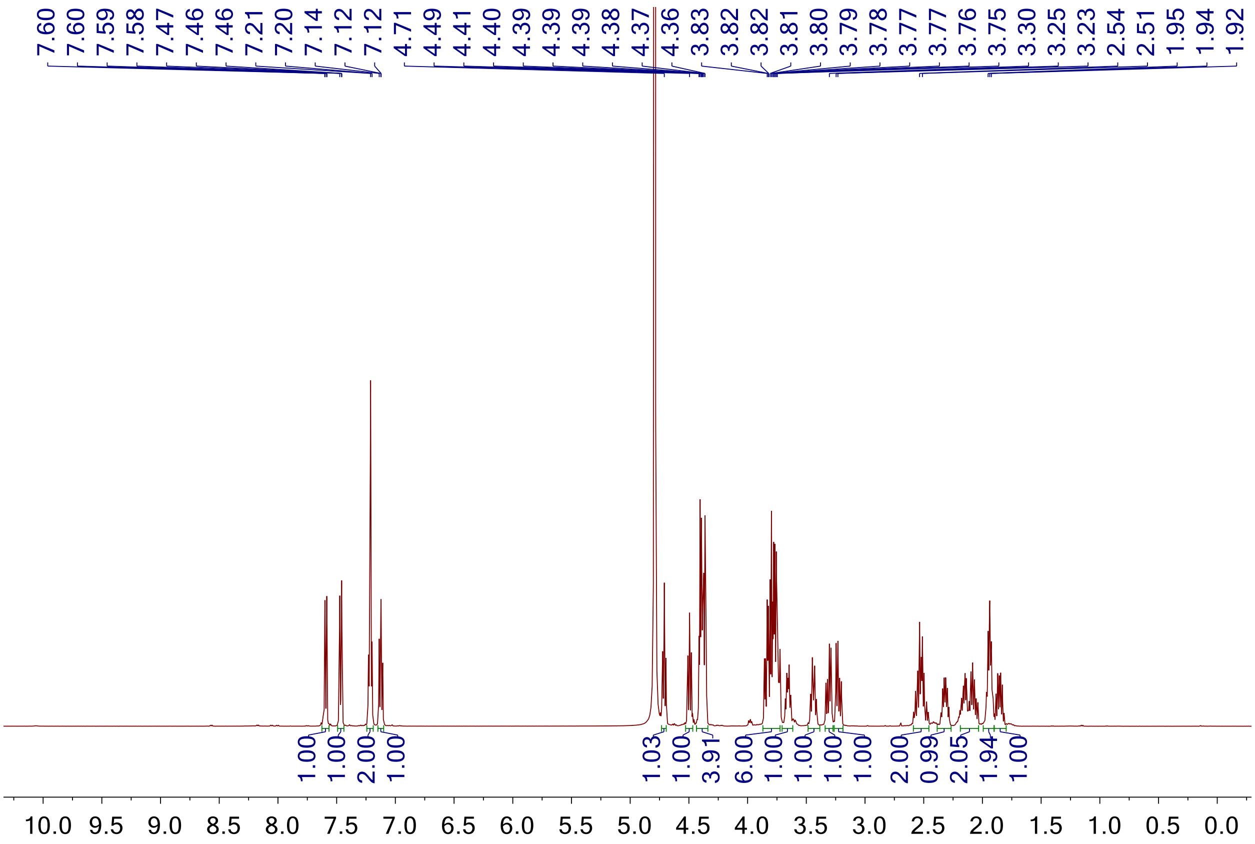

^{13}C NMR 500 MHz in D_2O measured at 298K

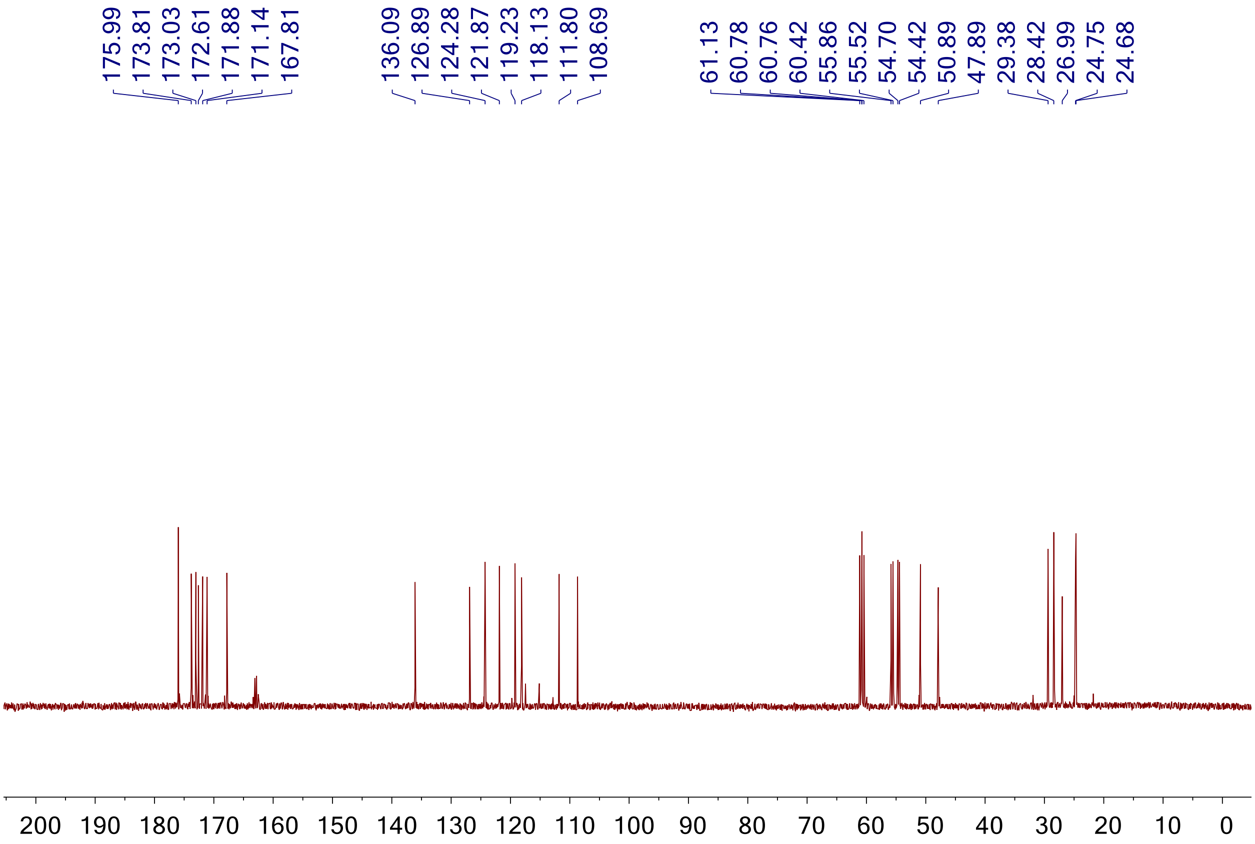

Supplementary Tables

Table S1. Potentially allosteric pockets predicted by PARS for hLang.

Pocket	Residues of hLang
1	W264, F272, K274, W281, P286
2	F202, K203, N205, Y207, S235, E238
3	H229, D268, K320, R321, P322

Table S2. Potentially allosteric pockets predicted by PARS for mLang.

Pocket	Residues of mLang
1	W267, F275, K277, S280, W284, P289
2	G201, W202, K203, Y210, Y211, F212,
3	N208, H232, D271, K323, R324, P325

Table S3. Peptides binding affinity for mLang and hLang.

Peptides	Structure	mLang/ K_D (µM)	hLang/ K_D (µM)
**AWS**	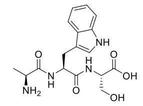	1600 ± 300	2400 ± 940
**SAWS**	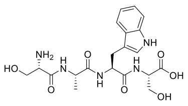	670 ± 230	820 ± 130
**EPSAWS**	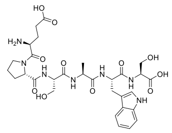	-	-
**SWS**	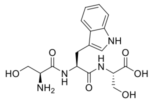	-	-
**SSWS**	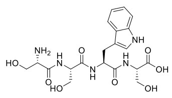	460 ± 30	-
**EPSSWS**	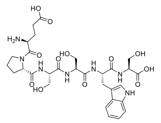	440 ± 100	-

Supplementary Figures

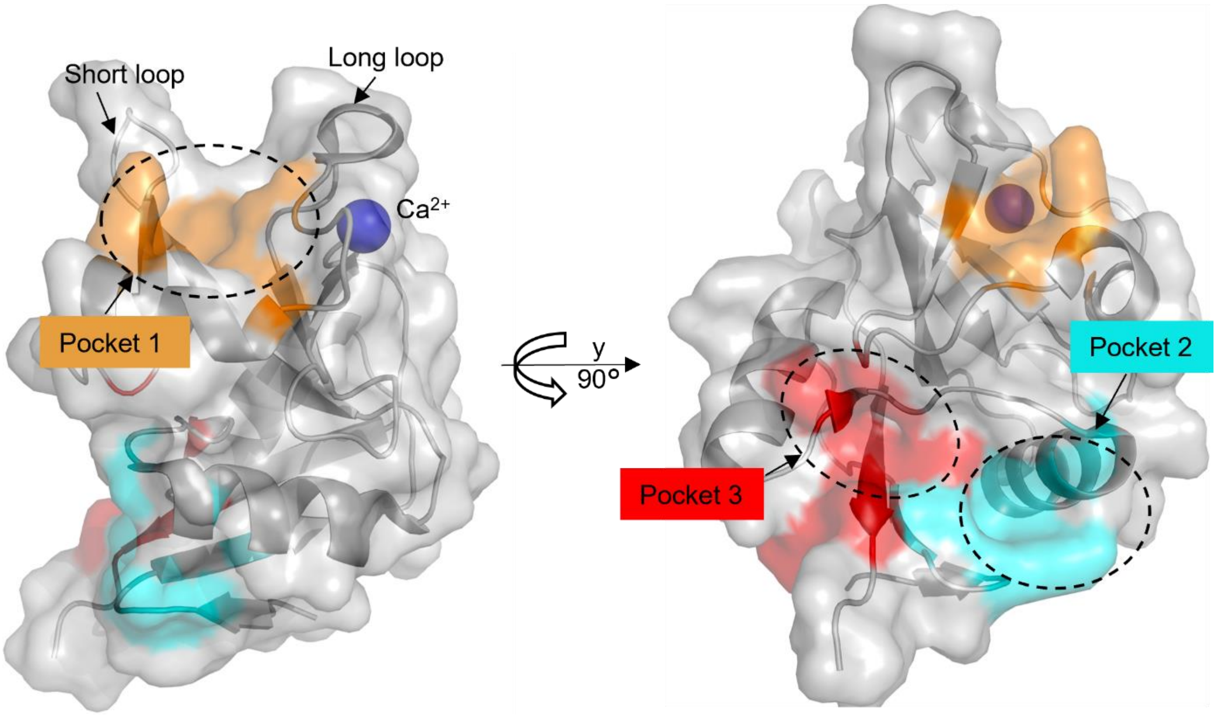

Figure S1. Potentially allosteric pockets predicted by PARS for hLang.

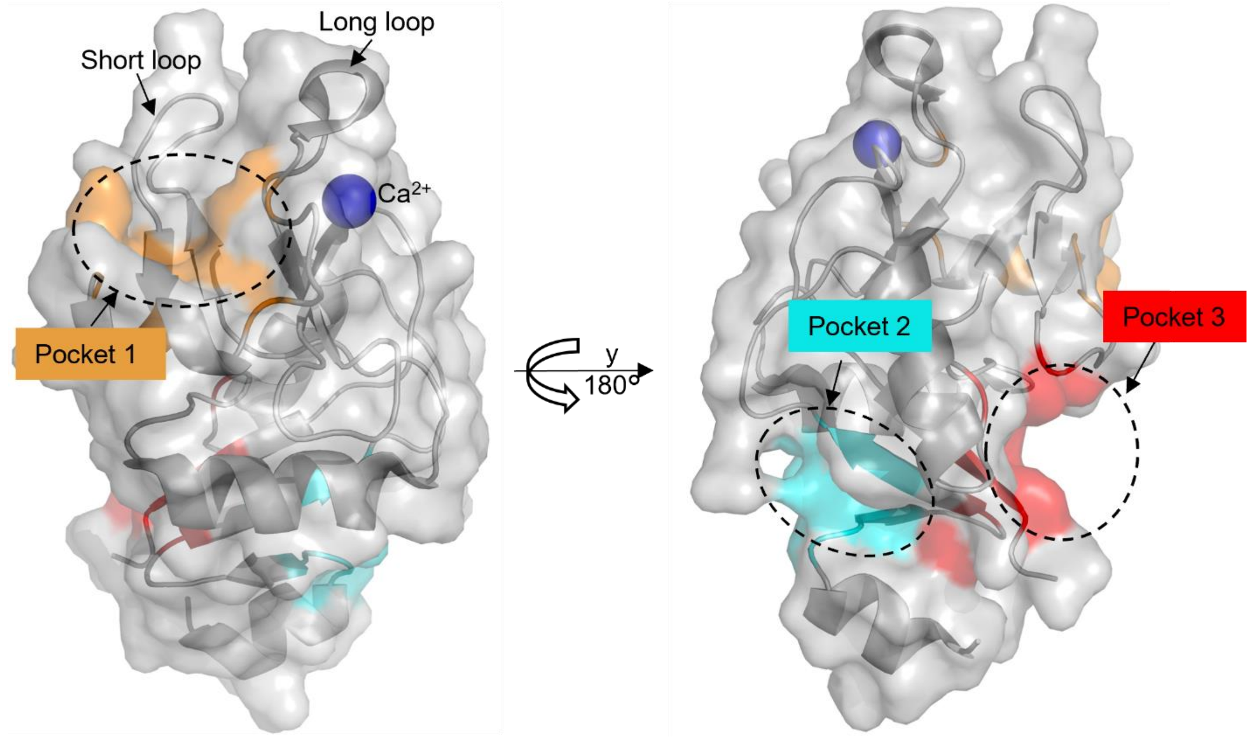

Figure S2. Potentially allosteric pockets predicted by PARS for mLang.

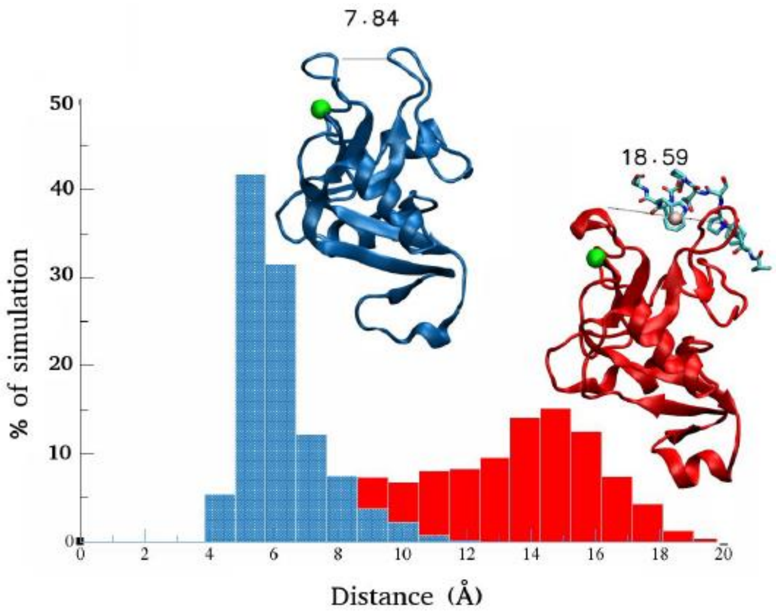

Figure S3. Measurement of distance between the short and long loop in mLang and hLang. Loop conformation analysis based on the distance between amide nitrogen of S263 and the carbonyl oxygen of N294 for mLang. Blue and red histograms were built from 1 microsecond of Molecular Dynamics Simulation starting from close or open modeled using the mLang structure (PDB number is 5M62) and hLang structure (PDB number is 3PH7), respectively. For each simulation, an illustrative structure (with SER-ASN distance in angstrom) for close/open simulation is shown in blue and red respectively. The green sphere represents calcium ion in the carbohydrate binding site.

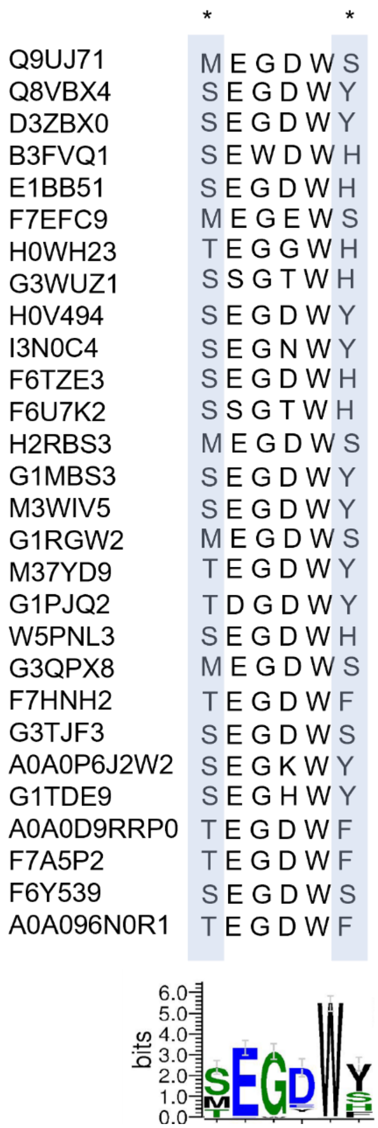

Figure S4. Multiple sequence alignment (MSA) and sequences conservation analysis in the short loop. MSA of 28 mammalian Langerin short loop sequences (upper). The short loop residues conservation degree based on MSA was analyzed by WebLogo (https://weblogo.berkeley.edu/logo.cgi) (below).

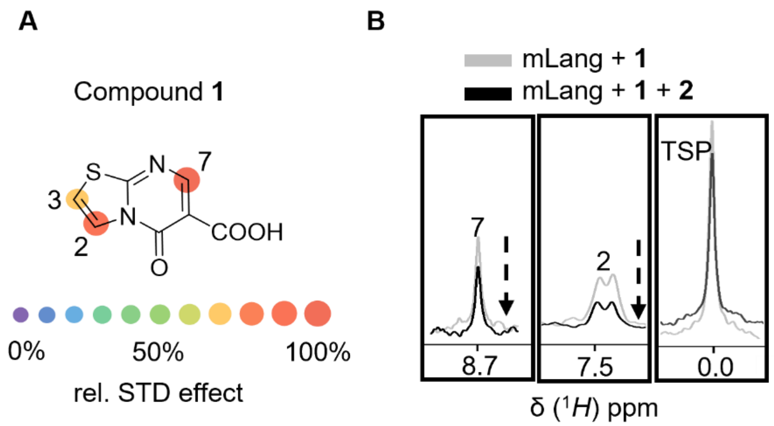

Figure S5. STD competition NMR experiments confirm that compound 1 and 2 are located in the same binding site. A. STD NMR epitope map of compound **1** and resonances H2 and H7 serve as indicator peaks during competition since the closer interaction with protein (saturation time is 2.0 s, relaxation time is 2.0 s, and the scans is 256). B. Competition NMR experiments exhibiting the compound **2** can compete with compound **1** in the same binding site. The STD NMR spectrum was recorded with 2 mM compound **1** in the presence of 40 μM of mLang ECD (grey). STD NMR spectrum of 300 μM compound **2** added into the mixture of **1** and the protein mixture is shown as a black line. This data indicates that the STD signal intensities of compound **1** (H2 and H7) were reduced after adding compound **2** (signal intensity reduced 40 - 50%).

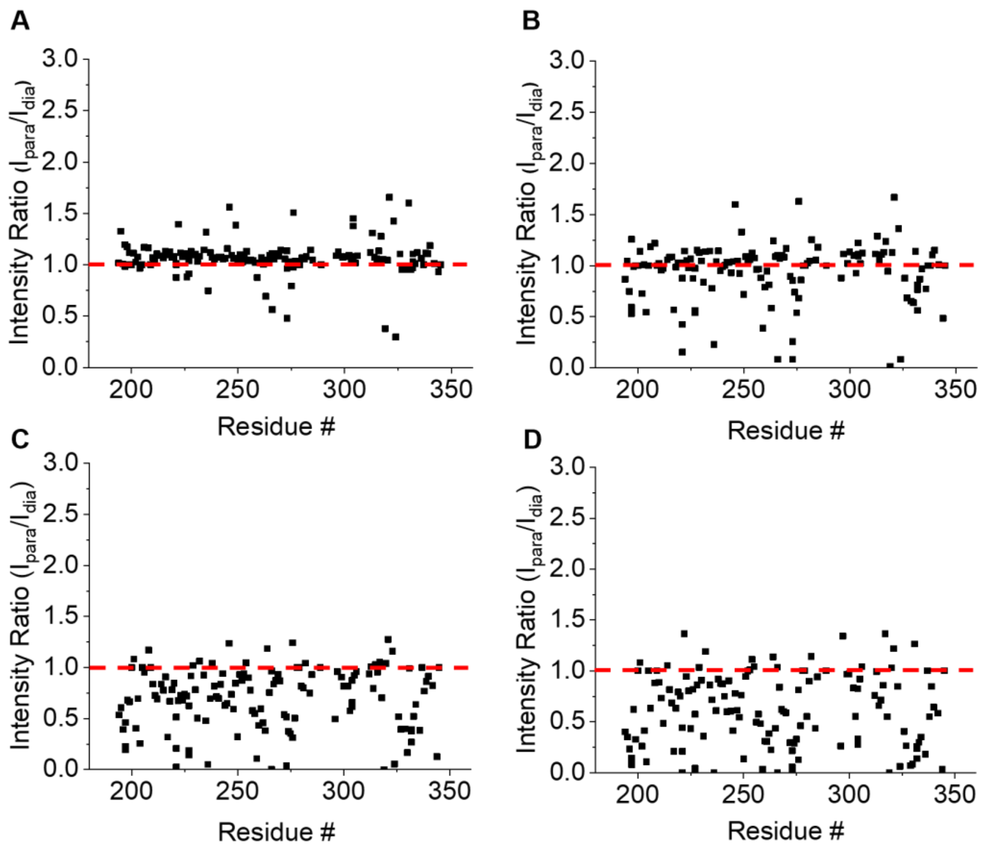

Figure S6. The intensity ratio of each amino acid residue between diamagnetic and paramagnetic effect of mLang CRD in the presence of a different concentration of TEMOL. A-D. The intensity ratio of each amino acid residues between diamagnetic and paramagnetic effect of mLang CRD in the presence of 1, 5, 15, and 25 mM TEMPOL, respectively. The red dash line means the intensity ratio is 1. 1 and 5 mM TEMPOL cannot induce enough paramagnetic effect and 25 mM generate too much paramagnetic effect to analyze. 15 mM TMEPOL was found to be the proper concentration for further analysis.

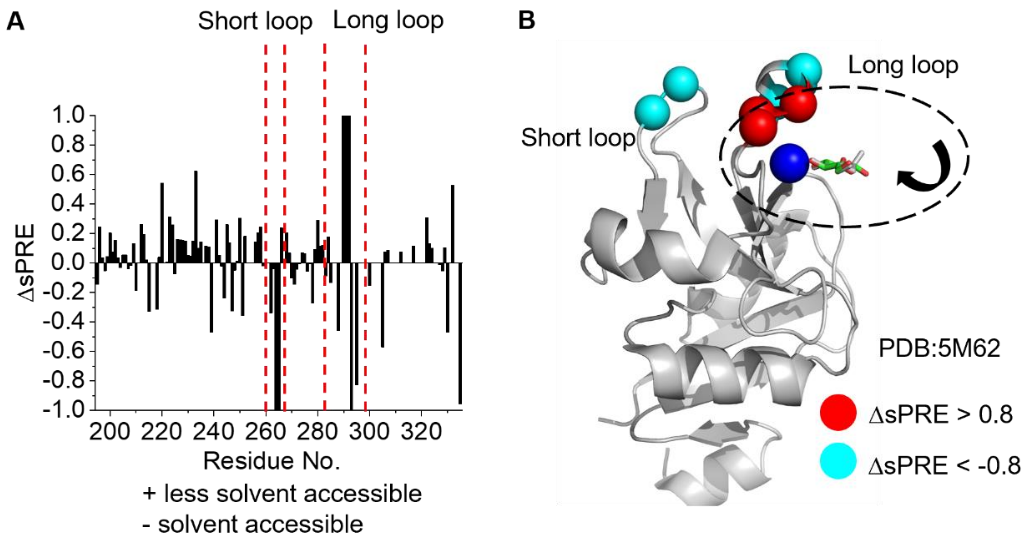

Figure S7. Mannose-binding site confirmation of mLang by solution paramagnetic relaxation enhancement (sPRE) experiments. Illustration of mannose bindings to mLangCRD. A. sPREs were obtained from the height values measured by using a ^{1}H–^{15}N HSQC-based NMR at concentrations of 15 mM of the soluble paramagnetic agent TEMPOL. The positive values in the histogram correspond to solvent inaccessible residues, indicating ligand binding, while negative values represent solvent accessibility after ligand binding. B. TEMPOL hot spot mapping on the mLang structure (PDB: 5M62). We tested mannose as an endogenous ligand. Mapping of the sPRE values on the X-ray structure of mLang revealed inaccessibility of residues in the CBS in proximity to the Ca^{2+} sites, such as the well characterized EPN motif residue N290. Additionally, highly solvent accessible residues were found to be located in the short (264E and G265) and long loop (G293 and N295), indicating dynamic structural changes mediated by mannose binding. The residues N290, N291 and A292 exhibiting ΔsPRE > 0.8 are labeled as red color spheres on the cartoon presentation of mLang. The residues E264 and G265 in the short loop and G293 and N295 in the long loop exhibiting ΔsPRE < -0.8 are labeled as blue color spheres on the cartoon presentation of mLang.

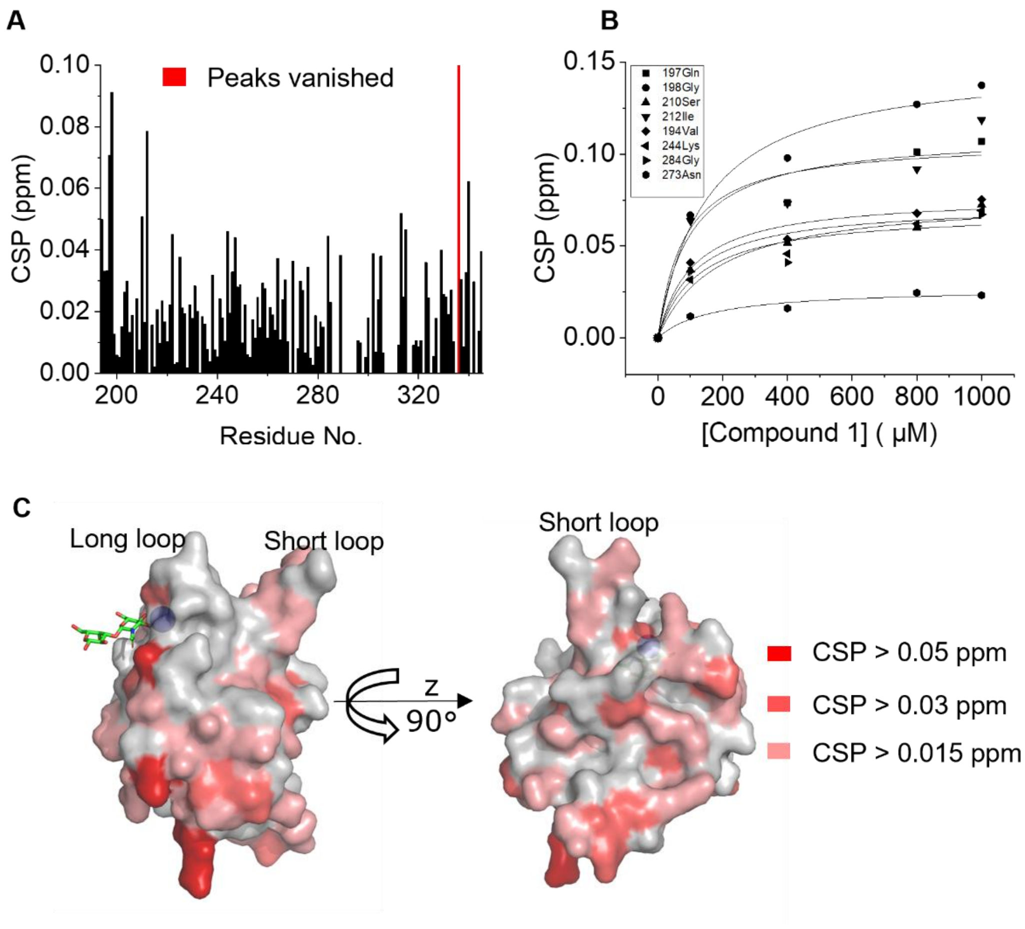

Figure S8. ^{15}N HSQC CSP fingerprint of compound 1 binding with hLang CRD wild type. A. CSP pattern observed in ^{15}N HSQC NMR confirms the involvement of residues in compound **1** binding with hLang CRD wild type. B. ^{15}N HSQC NMR titrations between hLang CRD wild type with compound **1**. Determination of affinity from CSP trajectories yielded K_D values for compound **1** (K_D = 130 ± 40 μM). C. Mapping of CSPs on the X-ray structure of hLang CRD (PDB: 3P7H).

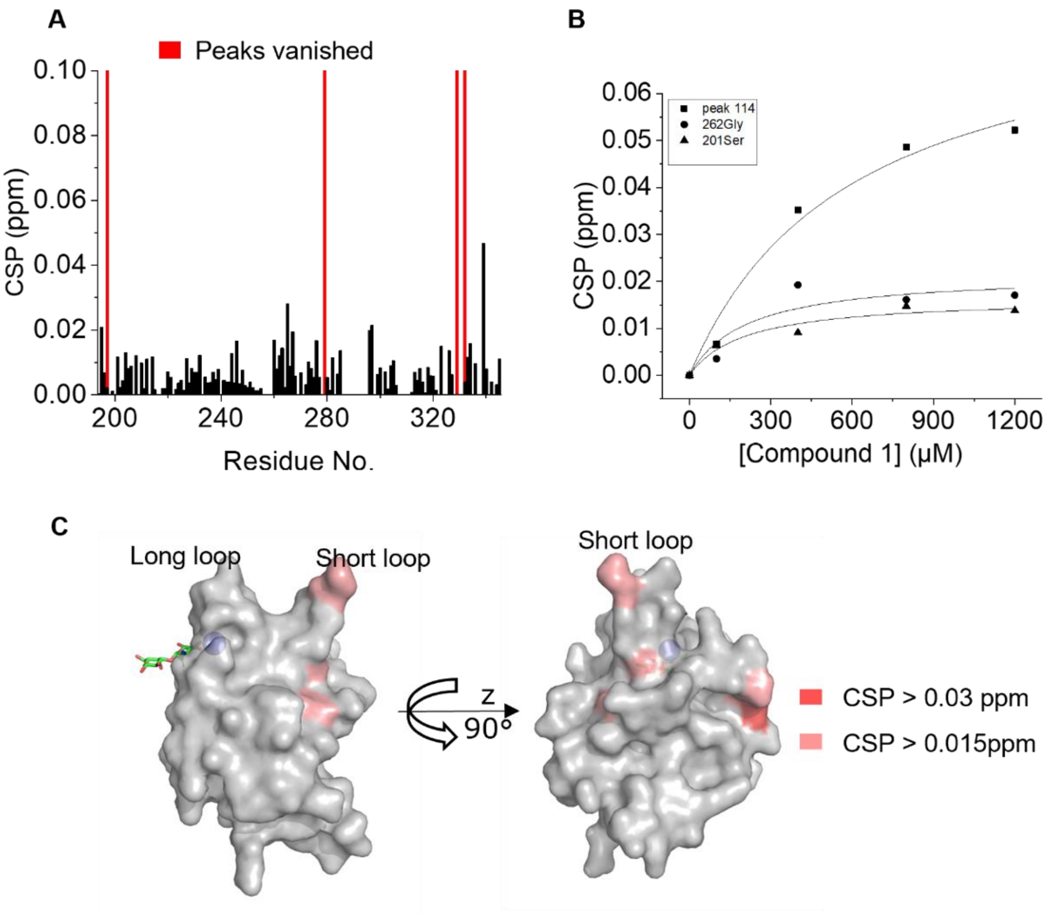

Figure S9. ^{15}N HSQC CSP fingerprint of compound 1 binding with hLang CRD-M260S-S265Y mutant. A. CSP pattern observed in ^{15}N HSQC NMR confirm involvement of residues in the compound **1** binding with hLang CRD-M260S-S265Y mutant. B. ^{15}N HSQC NMR titrations between hLang CRD M260S-S265Y mutant with compound **1**. Determination of affinity from CSP trajectories yielded K_D values for compound **1** (K_D = 340 ± 220 μM). C. Mapping of CSPs on the X-ray structure of hLang CRD (PDB: 3P7H).

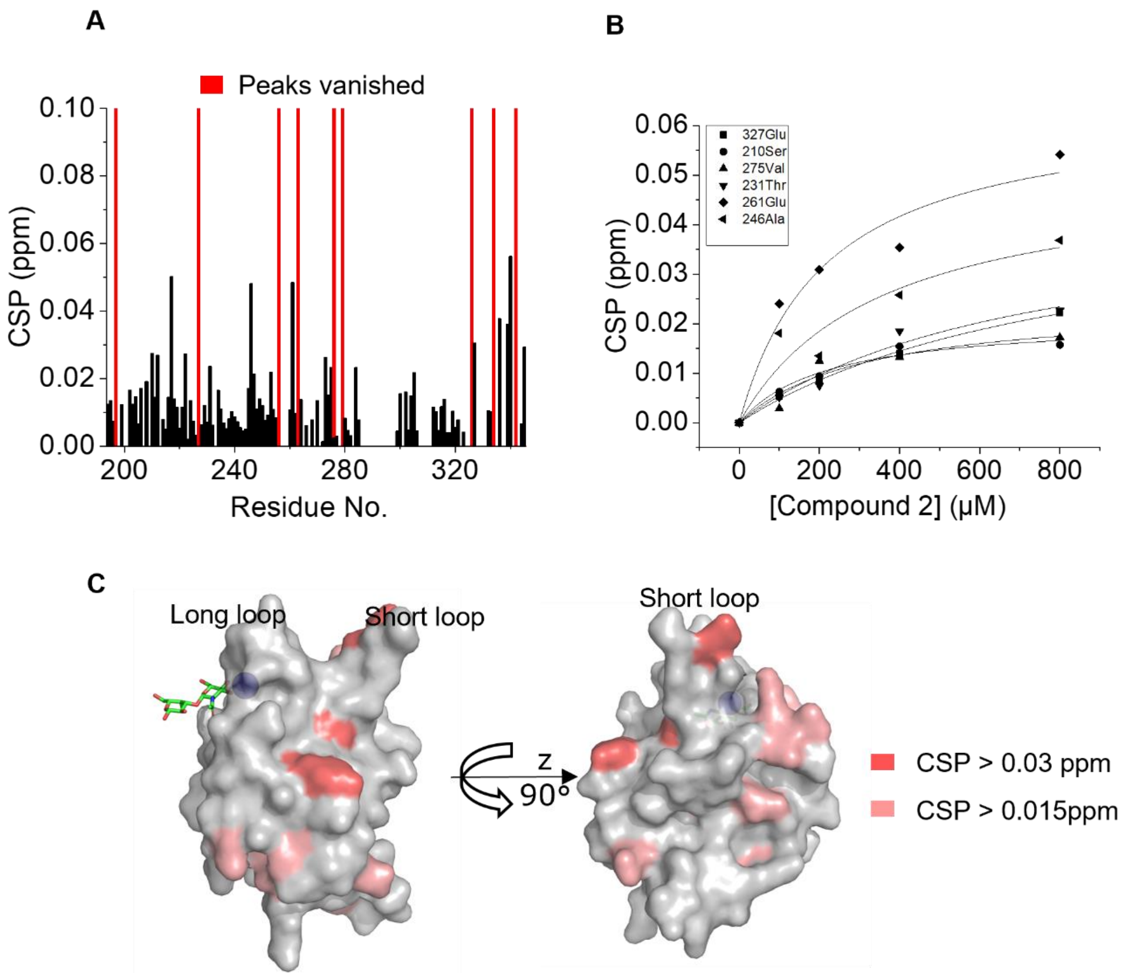

Figure S10. ^{15}N HSQC CSP fingerprint of compound 2 binding with hLang CRD-M260S-S265Y mutant. A. CSP pattern observed in ^{15}N HSQC NMR confirms the involvement of residues in the compound **2** binding with hLang CRD-M260S-S265Y mutant. B. ^{15}N HSQC NMR titrations between hLang CRD M260S-S265Y mutant with compound **2**. Determination of affinity from CSP trajectories yielded K_D values for compound **2** ($K_D = 450 \pm 280$ μM). C. Mapping of CSPs on the X-ray structure of hLang CRD (PDB: 3P7H).

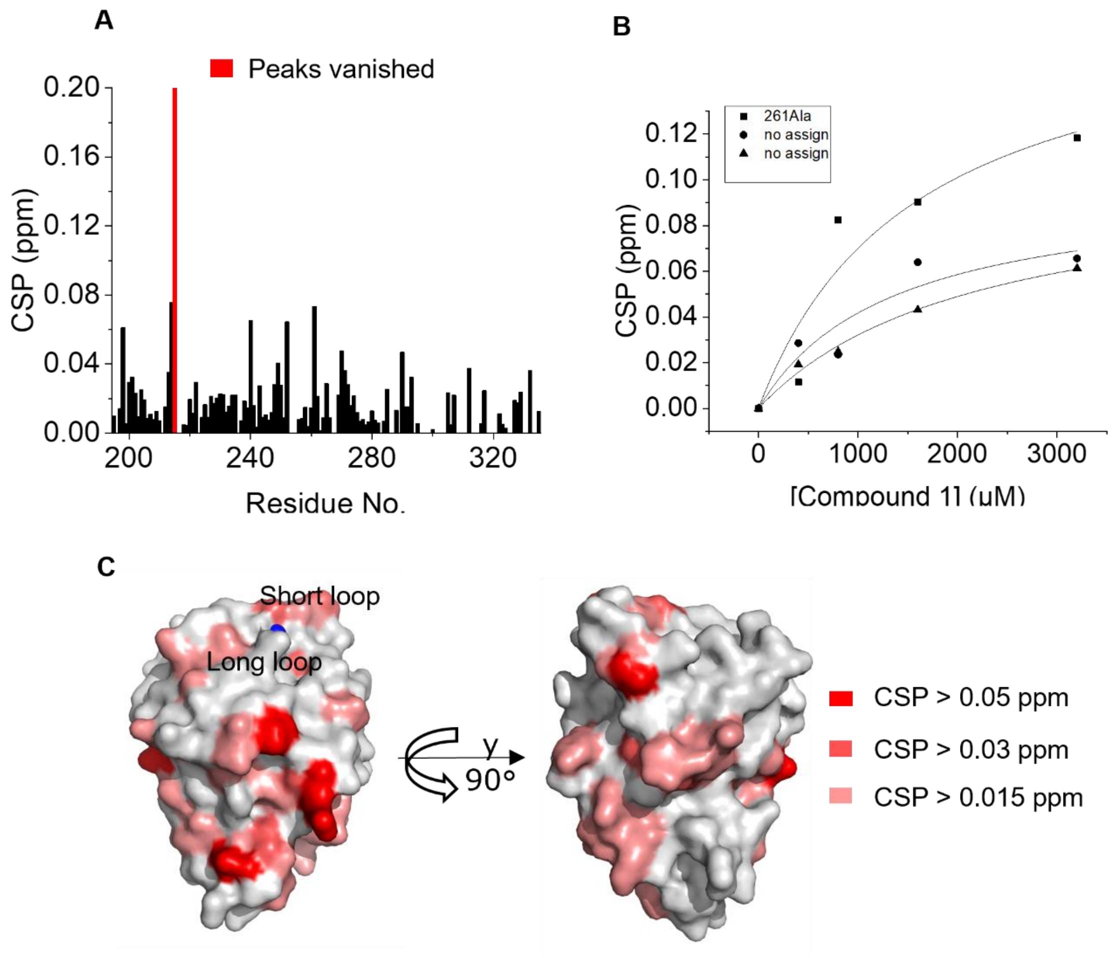

Figure S11. ^{15}N HSQC CSP fingerprint of compound 1 binding with mLang CRD wild type. A. CSP pattern observed in ^{15}N HSQC NMR confirms the involvement of residues in the compound **1** binding with mLang CRD wild type. B. ^{15}N HSQC NMR titrations between mLang CRD wild type with compound **1**. Determination of affinity from CSP trajectories yielded K_D values for compound 1 (K_D = 1700 ± 440 µM). C. Mapping of CSPs on the X-ray structure of mLang CRD (PDB: 5K8Y).

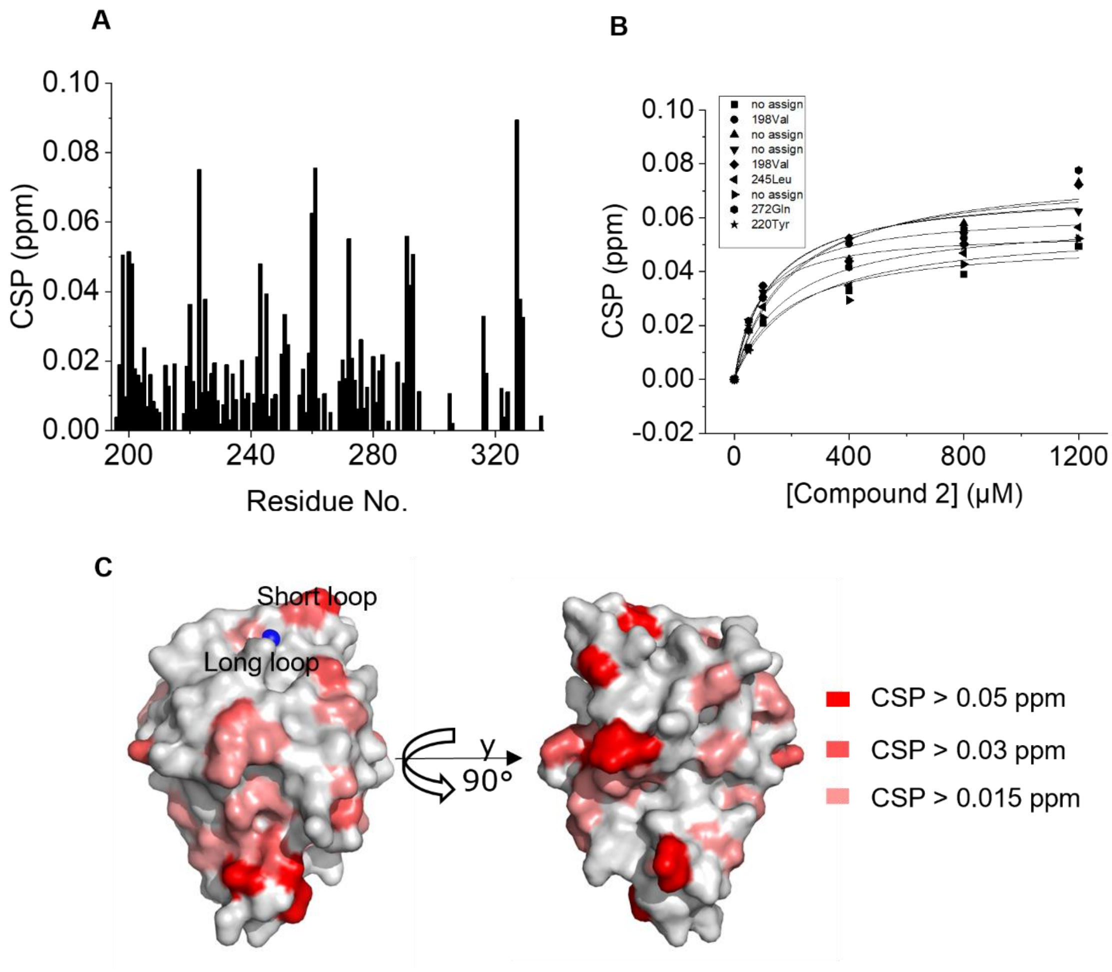

Figure S12. ^{15}N HSQC CSP fingerprint of compound 2 binding with mLang CRD wild type. A. CSP pattern observed in ^{15}N HSQC NMR confirms the involvement of residues in the compound **2** binding with mLang CRD wild type. B. ^{15}N HSQC NMR titrations between mLang CRD wild type with compound **2**. Determination of affinity from CSP trajectories yielded K_D values for compound **2** (K_D = 160 ± 60 μM). C. Mapping of CSPs on the X-ray structure of mLang CRD (PDB: 5K8Y).

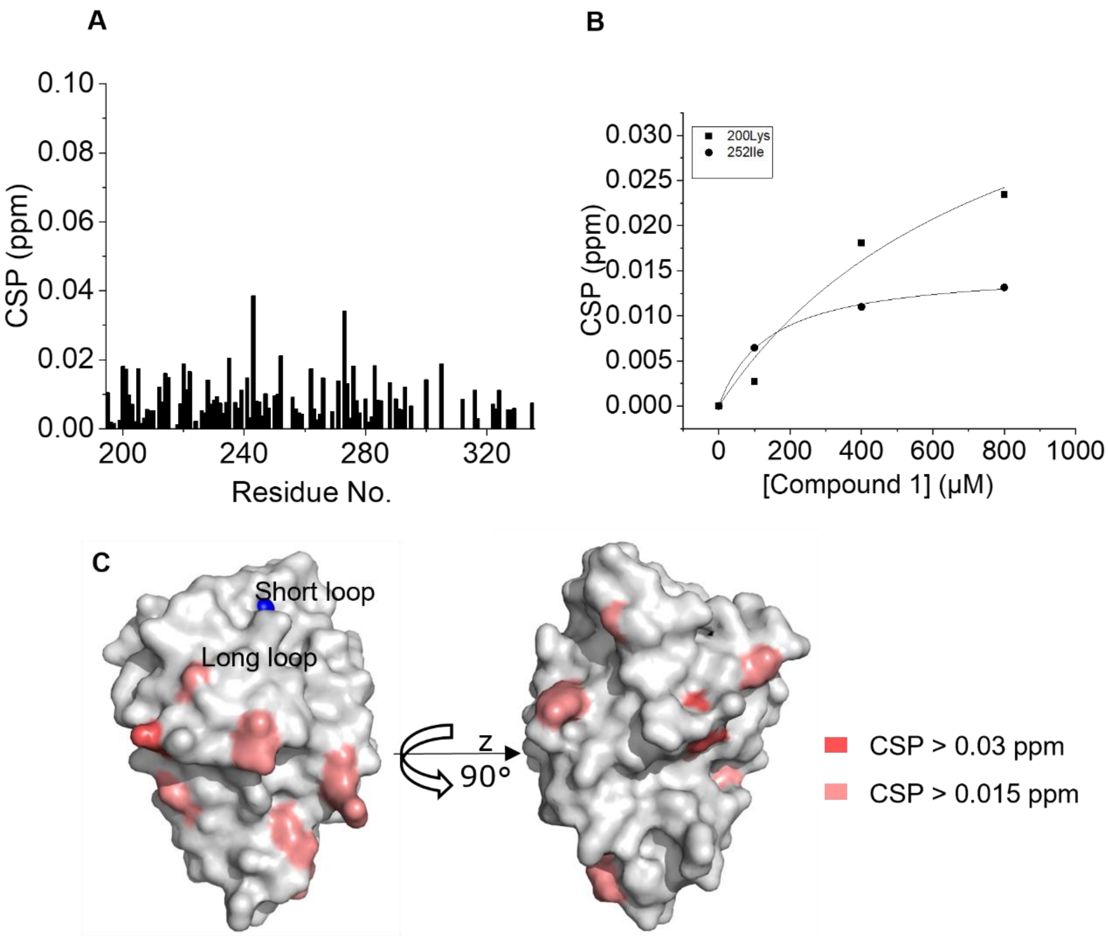

Figure S13. ^{15}N HSQC CSP fingerprint of compound 1 binding with mLang CRD-S263M-Y268S. A. CSP pattern observed in ^{15}N HSQC NMR confirms the involvement of residues in the compound **1** binding with mLang CRD-S263M-Y268S. B. ^{15}N HSQC NMR titrations between mLang CRD-S263M-Y268S with compound **1**. Determination of affinity from CSP trajectories yielded K_D values for compound **1** (K_D = 480 ± 340 μM). C. Mapping of CSPs on the X-ray structure of mLang CRD (PDB: 5K8Y).

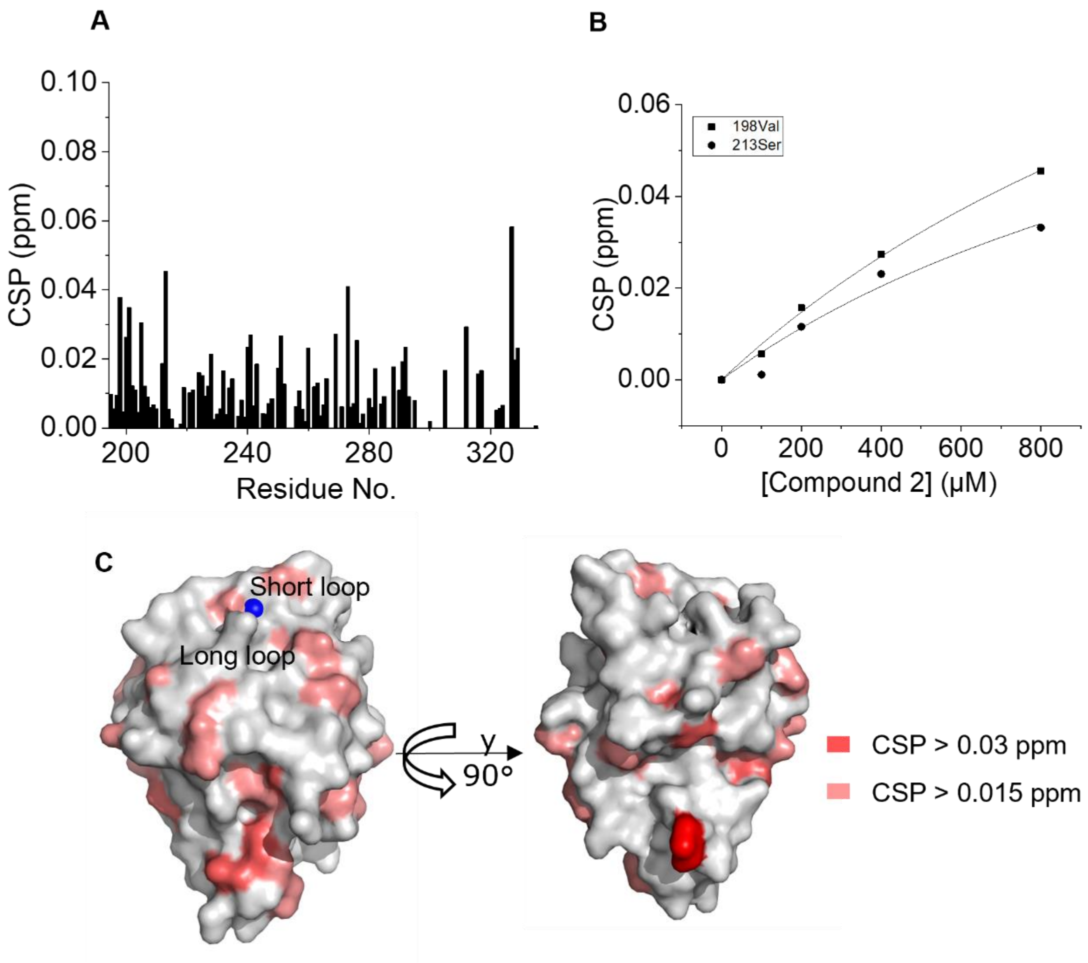

Figure S14. ^{15}N HSQC CSP fingerprint of compound 2 binding with mLang CRD-S263M-Y268S. A. CSP pattern observed in ^{15}N HSQC NMR confirms the involvement of residues in the compound **2** binding with mLang CRD-S263M-Y268S. B. ^{15}N HSQC NMR titrations between mLang CRD-S263M-Y268S with compound **2**. Determination of affinity from CSP trajectories yielded K_D values for compound 2 (K_D = 1740 ± 160 μM). C. Mapping of CSPs on the X-ray structure of mLang CRD (PDB: 5K8Y).

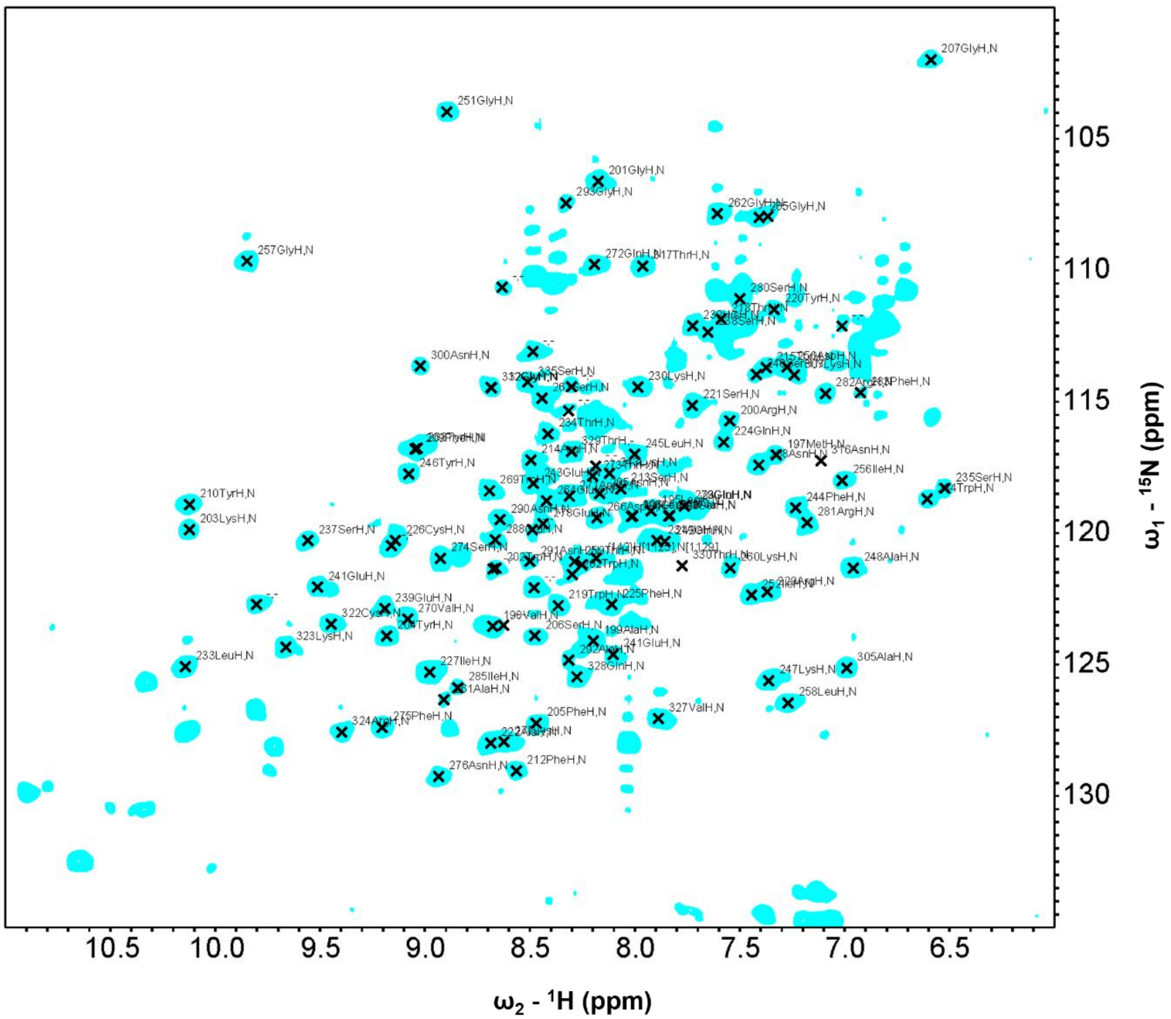

Figure S15. ^{1}H-^{15}N HSQC NMR spectrum with assigned backbone and side-chain resonances of recombinant mLang CRD construct His tag. 88% of all backbone amide resonances were assigned using standard triple resonance spectra and NOESY spectra. All assignment spectra were recorded at pH 6, 298 K, and in presence of 5 mM $CaCl_2$ in MES buffer.

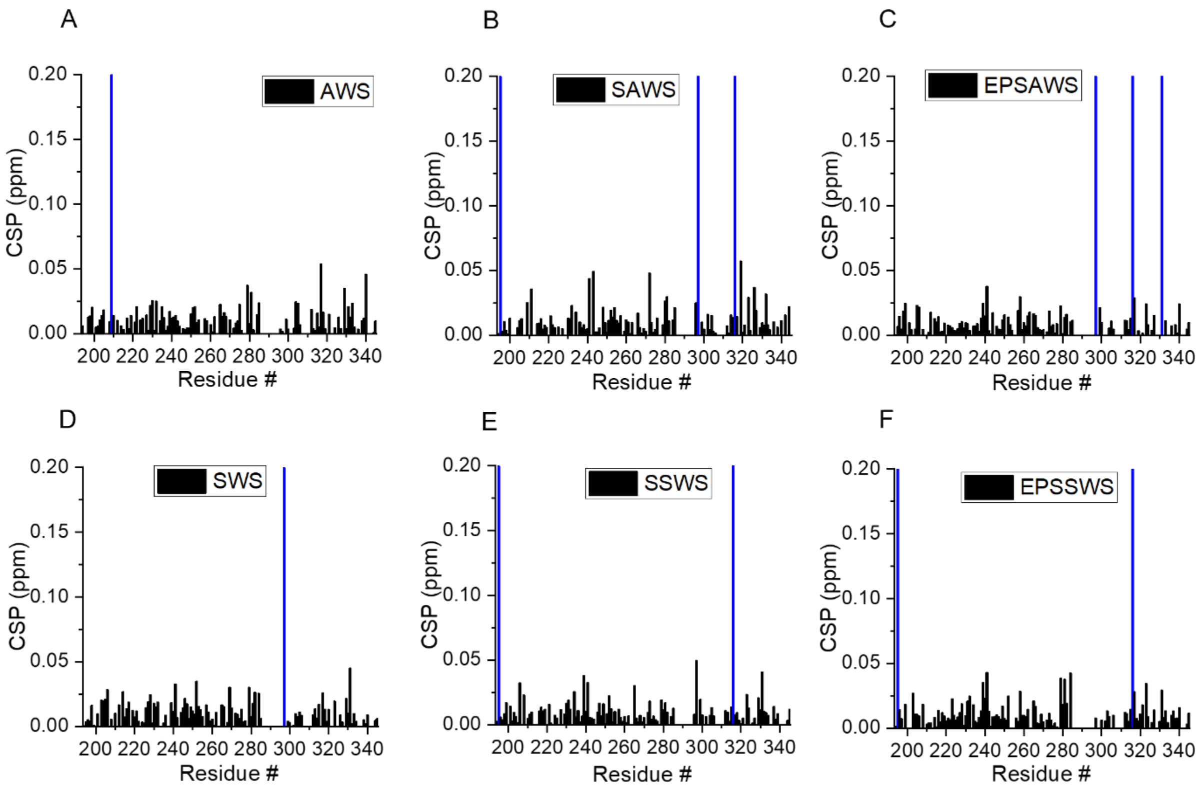

Figure S16. CSPs of 1 mM peptides binding with hLang CRD WT. A-F. CSP plots of 1 mM peptide **AWS**, **SAWS**, **EPSAWS**, **SWS**, **SSWS**, and **EPSSWS** with hLang CRD WT, respectively. Peaks vanished upon peptide binding are highlighted in blue.

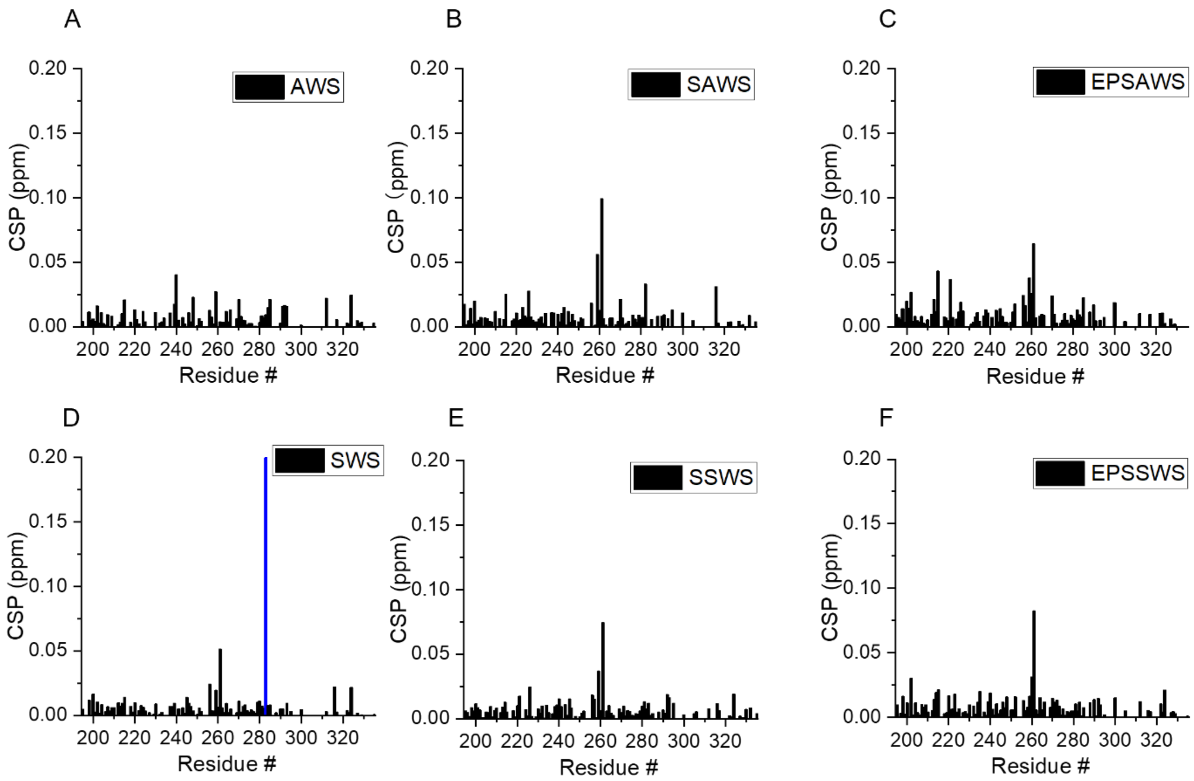

Figure S17. CSPs of 1mM peptides binding with mLang CRD WT. A-F. CSP plots of 1 mM peptide **AWS**, **SAWS**, **EPSAWS**, **SWS**, **SSWS**, and **EPSSWS** with mLang CRD WT, respectively. Peaks vanished upon peptide binding are highlighted in blue.

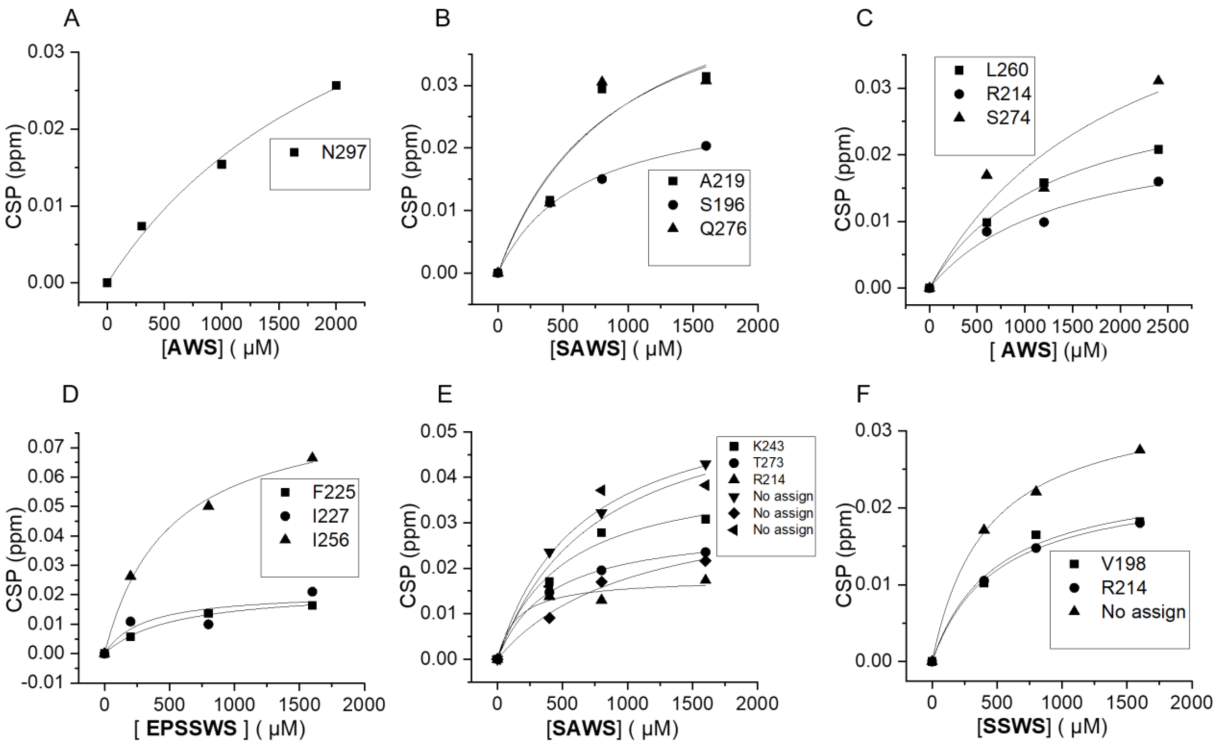

Figure S18. Binding affinity of peptides to hLang and mLang by ^{15}N HSQC NMR. A. ^{15}N HSQC NMR titrations between hLang CRD-WT with AWS. Determination of affinity from CSP trajectories yielded K_D values for AWS (K_D = 2400 ± 940 μM). B. ^{15}N HSQC NMR titrations between hLang CRD-WT with SAWS. Determination of affinity from CSP trajectories yielded K_D values for SAWS (K_D = 820 ± 130 μM). C. ^{15}N HSQC NMR titrations between mLang CRD-WT with AWS. Determination of affinity from CSP trajectories yielded K_D values for AWS (K_D = 1600 ± 300 μM). D. ^{15}N HSQC NMR titrations between mLang CRD-WT with EPSSWS. Determination of affinity from CSP trajectories yielded K_D values for EPSSWS (K_D = 440 ± 100 μM). E. ^{15}N HSQC NMR titrations between mLang CRD-WT with SAWS. Determination of affinity from CSP trajectories yielded K_D values for SAWS (K_D = 670 ± 230 μM). D. ^{15}N HSQC NMR titrations between mLang CRD-WT with SSWS. Determination of affinity from CSP trajectories yielded K_D values for SSWS (K_D = 460 ± 30 μM).

References

(1) Hanske, J.; Aleksić, S.; Ballaschk, M.; Jurk, M.; Shanina, E.; Beerbaum, M.; Schmieder, P.; Keller, B. G.; Rademacher, C. (2016) Intradomain Allosteric Network Modulates Calcium Affinity of the C-Type Lectin Receptor Langerin. *J. Am. Chem. Soc. 138* (37), 12176-12186.

(2) Vranken, W. F.; Boucher, W.; Stevens, T. J.; Fogh, R. H.; Pajon, A.; Llinas, M.; Ulrich, E. L.; Markley, J. L.; Ionides, J.; Laue, E. D. (2005) The CCPN data model for NMR spectroscopy: development of a software pipeline. *Proteins 59* (4), 687-696.

(3) Blanco Capurro, J. I.; Di Paola, M.; Gamarra, M. D.; Martí, M. A.; Modenutti, C. P. (2018) An efficient use of X-ray information, homology modeling, molecular dynamics and knowledge-based docking techniques to predict protein–monosaccharide complexes. *Glycobiology 29* (2), 124-136.

(4) Maier, J. A.; Martinez, C.; Kasavajhala, K.; Wickstrom, L.; Hauser, K. E.; Simmerling, C. (2015) ff14SB: Improving the Accuracy of Protein Side Chain and Backbone Parameters from ff99SB. *J. Chem. Theory. Comput. 11* (8), 3696-3713.

(5) Hopkins, C. W.; Le Grand, S.; Walker, R. C.; Roitberg, A. E. (2015) Long-Time-Step Molecular Dynamics through Hydrogen Mass Repartitioning. *J. chem. theory comput. 11* (4), 1864-1874.

(6) Roe, D. R.; Cheatham, T. E. (2013) PTRAJ and CPPTRAJ: Software for Processing and Analysis of Molecular Dynamics Trajectory Data. *J. chem. theory comput. 9* (7), 3084-3095.

(7) Aretz, J.; Anumala, U. R.; Fuchsberger, F. F.; Molavi, N.; Ziebart, N.; Zhang, H.; Nazaré, M.; Rademacher, C. (2018) Allosteric Inhibition of a Mammalian Lectin. *J. Am. Chem. Soc. 140* (44), 14915-14925.
